# Double‐Sided Mechanical Interlocking Enables Soft‐Rigid Conductive Interfaces With a Record High Toughness for Flexible Electronics

**DOI:** 10.1002/adma.73649

**Published:** 2026-06-09

**Authors:** Gang Li, Minkun Cai, Chunyan Cao, Zengbai Ouyang, Hong Fu, Lingyu Zhao, Bingang Xu

**Affiliations:** ^1^ Nanotechnology Center, School of Fashion and Textiles The Hong Kong Polytechnic University Hong Kong China; ^2^ Research Centre for Resources Engineering Towards Carbon Neutrality The Hong Kong Polytechnic University Hong Kong China; ^3^ Department of Materials Science and Engineering Southern University of Science and Technology Shenzhen China; ^4^ School of Electrics and Computer Engineering Nanfang College Guangzhou Guangzhou China; ^5^ Department of Mathematics and Information Technology The Education University of Hong Kong Hong Kong China

**Keywords:** conductive polymer, flexible electronics, mechanical interlock, soft‐rigid interface, thread‐hole

## Abstract

The pronounced mismatch between polymeric electrodes and metallic components hinders the formation of robust electrical contacts. While most approaches rely on chemical design to strengthen interfacial interactions, we present a double‐sided mechanical interlocking strategy that provides both stability and adaptability. A conductive fabric scaffold bridges polymers and metals, with adhesives sequentially applied to both sides. The adhesive infiltrates and encapsulates scaffold fibers, forming a thread–hole adhesion that can only be disrupted by bulk failure. This mechanism achieves a record high interfacial toughness of 730 J m^−^^2^ between conductive elastomer and copper using commercial silver pastes. Peeling tests show delamination occurs between silver paste and copper, indicating even higher toughness could be obtained with better‐performing products of conductive adhesive. Notably, the interface stability surpasses that of the electrode itself, remaining intact even when the electrode fails. The design is broadly compatible with elastomeric or hydrogel matrices and with diverse commercial adhesives. It enables the construction of reliable epidermal electronics and hydrogel‐based devices. Overall, this interlocking strategy provides a versatile platform for integrating soft and rigid conductors in hybrid electronic systems.

## Graphical abstract

A double‐sided mechanical interlocking strategy is developed to create robust electrical contact between polymer electrode and metal interconnect. The fibrous structure enables formation of thread–hole adhesion, which only breaks under bulk failure and achieves a record high interfacial energy exceeding 730 J·m^−^^2^. This adhesion secures the integrity of interface under extreme strain, which may be vital for flexible electronics.

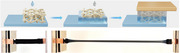

## 1 Introduction

**FIGURE 1:**
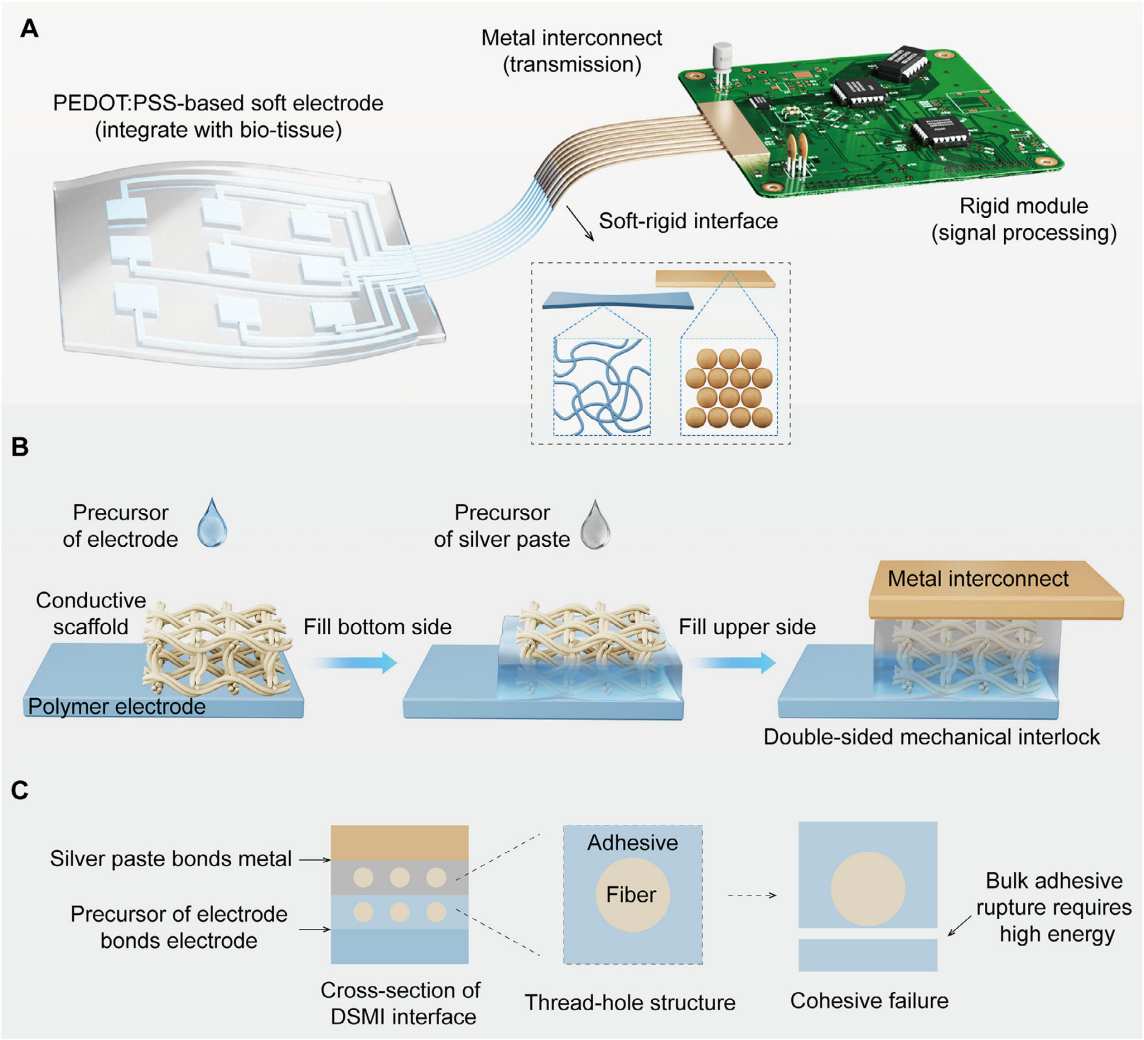
Illustration of the soft‐rigid interface and the design of the DSMI connection. (A) A flexible electronics device composed of a soft module with a conductive polymer‐based electrode for better integration with biotissue, and a rigid module for signal processing. A soft‐rigid interface forms when the signal is transmitted through these two components. (B) A two‐step procedure for DSMI‐connection fabrication. (C) The cross‐section of the DSMI interface reveals the feature of a thread‐hole type mechanical interlocking.

Flexible electronic devices employ soft polymeric conductors, most notably poly(3,4‐ethylenedioxythiophene):poly(styrenesulfonate) (PEDOT:PSS), to fabricate functional sensing elements that enable seamless bio‐integration for health monitoring and treatment [1, 2, 3, 4, 5, 6, 7, 8, 9, 10, 11]. However, these systems inevitably depend on rigid semiconductors and metallic interconnects for signal processing, acquisition, and transmission (Figure 1A). This hybrid configuration results in continuous electronic signal transmission across the soft PEDOT:PSS‐based electrode and the rigid metallic conductor interfaces. The fundamental operational requirement thus inherently creates mechanical incompatibility. The large disparity in mechanical properties, including elastic modulus and strain response between polymeric conductors and their rigid counterparts, initiates progressive interfacial delamination at soft‐rigid junctions under strain, ultimately resulting in electrical failure and systemic device degradation [12, 13, 14].

Conventional interconnect technologies that rely on conductive adhesives, such as silver pastes (SP) and anisotropic conductive films (ACF), are optimized for rigid metallic conductors with high surface energies (≈1500 mN m^−1^ for Au and ≈1800 mN m^−1^ for Cu) [15]. In contrast, PEDOT:PSS‐based electrodes typically have low surface energy (≈70 mN m^−1^ for pure PEDOT:PSS [16]). Moreover, SP and ACF use a hydrophobic resin matrix, whereas most PEDOT:PSS‐based electrodes are hydrophilic. Given these disparities between PEDOT:PSS‐based materials and metals, designing a single conductive adhesive that adheres well to both is challenging; consequently, using adhesives engineered for metals directly on PEDOT:PSS (Table S1) often yields low interfacial toughness, failing to ensure reliable mechanical integrity and stable electrical conductivity under mechanical strain.

Emerging interconnect technologies are broadly divided into material‐centric and structural strategies. Material‐centric designs focus on enhancing interfacial bonding through chemical engineering [12, 13, 14, 17, 18, 19, 20, 21, 22]. For instance, Jiang et al. developed a biphasic nano‐dispersed interface by embedding gold or silver nanoparticles within an elastomer matrix. This nanostructure facilitates rapid, paste‐free assembly between soft and rigid modules via matrix self‐adhesion and electrical percolation pathways [12]. Similarly, Ai et al. engineered a supramolecular confined liquid metal composite that exploits interfacial hydrogen‐bonding motifs to achieve robust adhesion to diverse substrates while maintaining conductivity under deformation [20]. However, these material‐centric approaches were developed for metal‐based systems and cannot be directly applied to PEDOT:PSS‐based conductive elastomers or hydrogels [5, 23, 24, 25, 26], primarily due to their low surface energy and hydrophilicity, as mentioned above.

Complementary structural strategies aim to mitigate interfacial strain through geometric design rather than chemical engineering [13, 21]. Zhuang et al. demonstrated that using a porous substrate with liquid metal as the conductive medium can provide electrical connections between component pins and stretchable circuits under large strain, enabling highly deformable electronic devices [27]. However, while the liquid metal's fluidity supports stable conductivity, the mechanical stability of the interface remains questionable. Tang et al. developed an “e‐vest” approach that encapsulates rigid electronics in a vest; the vest's insulating regions bond to a stretchable substrate via mechanical interlocking, thereby enabling stretchable systems [28]. Nonetheless, their conductive interconnects still rely on liquid metal and tin solder. Thus, there remains an urgent need for a robust method to form reliable conductive interfaces between PEDOT:PSS‐based electrodes and metal interconnects to realize durable flexible electronics.

Herein, we propose a double‐sided mechanical interlocking (DSMI) strategy for reliable soft‐rigid conductive interfaces (Figure 1B). We employ a conductive porous fibrous scaffold as an interfacial bridge. The scaffold's open‐pore architecture enables adhesive precursor infiltration, forming thread‐hole interlocks upon curing. Through a subsequent application, one scaffold surface bonds to the elastomer using the precursor of the elastomer while the opposite surface connects to metallic conductors via commercial silver paste (Figure 1C). This dual‐sided interlock achieves robust electrical and mechanical coupling between soft and rigid components. The interface maintains functionality under cyclic stretch deformation and significantly reduces motion artifacts in epidermal electrophysiological sensing. Furthermore, the thread‐hole adhesion remains effective during polymer hydration, enabling reliable hydrogel‐metal interconnects for hydrogel bioelectronics. This approach eliminates new adhesive development, accommodates both dry elastomers and wet hydrogels, and maintains compatibility with standard interconnection methods, thus providing a versatile platform for advanced hybrid electronics.

## 2 Results and Discussion

### 2.1 Design Principle

Mechanical interlocking enhances adhesion through structural design and, depending on the topology, can be classified into key‐lock and thread‐hole types. For soft‐rigid interfaces in flexible electronics, these two architectures exhibit markedly different performance. Because soft materials readily deform under external forces, the anchoring effect provided by key‐lock structures is limited, and interfacial stability still heavily relies on intrinsic interfacial interactions [29]. In contrast, in thread‐hole architectures, interfacial separation inevitably leads to bulk fracture of one of the components, and the energy dissipated during this process is directly related to the material's intrinsic toughness. This shift in failure mode enables thread‐hole structures to achieve robust interfacial stability even without relying on specific interfacial chemistry (Section S1 and Figure S1). Thus, we hypothesize that the thread‐hole design could support robust soft‐rigid connections in flexible electronics without the need to develop new conductive adhesives.

### 2.2 Mechanical and Electrical Performance of DSMI Connection

We used a 60‐count cotton fabric scaffold to establish the DSMI‐connection at soft‐rigid conductor interfaces. The fabric was first dip‐coated in PEDOT:PSS solution to transform into a conductive scaffold (CS) (Figure S2). Then we selected a conductive elastomer based on PEDOT:PSS and waterborne polyurethane (WPU) as a representative soft conductor (Figure S3) to study its connection with a typical rigid conductor of copper foil. This conductive scaffold was then integrated via sequential adhesive application: initially placed on the PEDOT:PSS/WPU conductive elastomer, followed by deposition and curing of the PEDOT:PSS/WPU precursor solution as the conductive adhesive to bond the scaffold onto the soft electrode. Commercial SP was subsequently applied, and copper foil was laminated onto the SP‐coated surface before final curing (Figure S4). For comparison, control interfaces were prepared using ACF and direct SP bonding between identical soft electrodes and copper foil.

**FIGURE 2:**
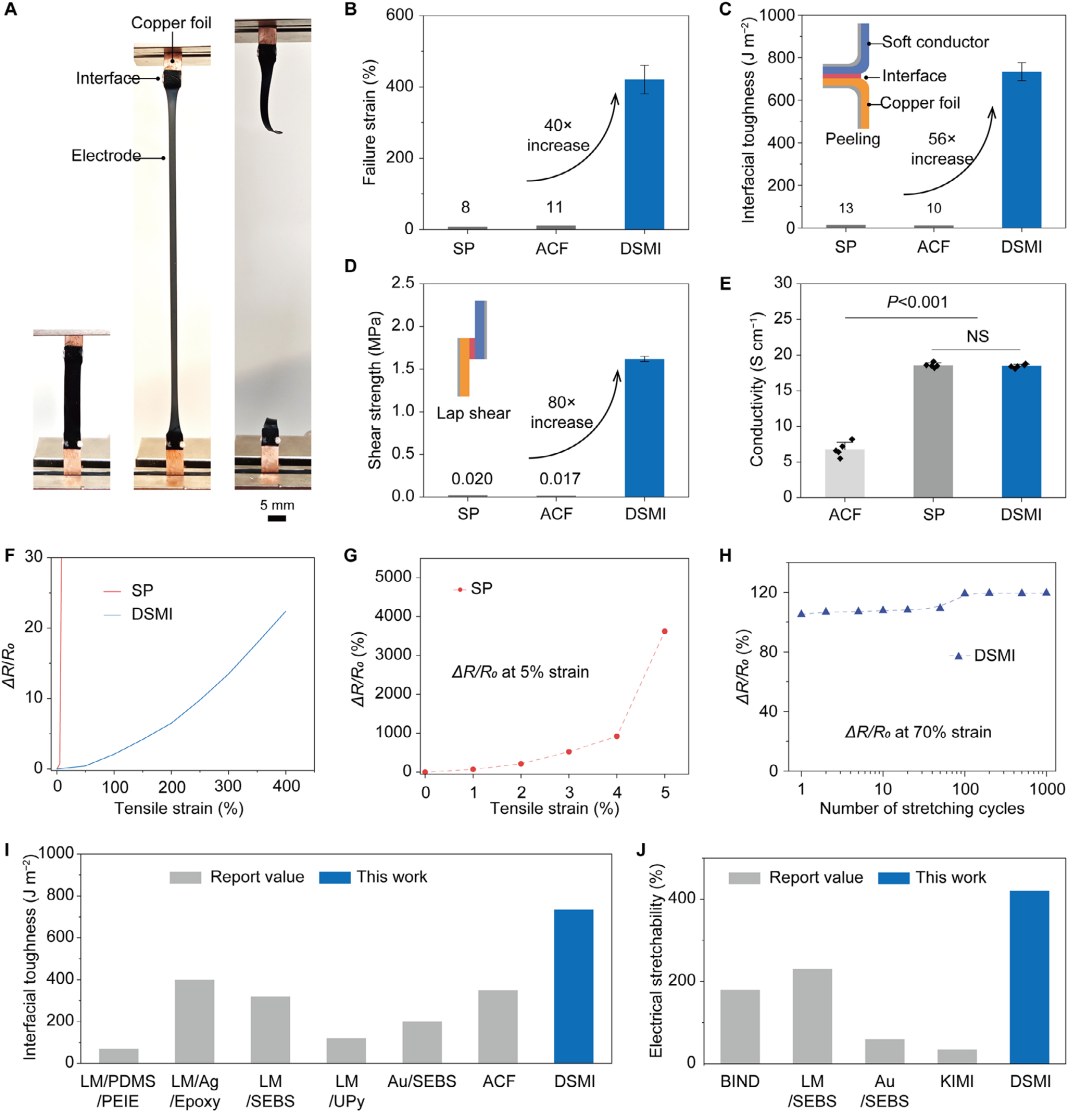
Performance of the DSMI connection. (A) A copper foil is connected to a polymer electrode via DSMI; during stretching until fracture of the polymer electrode, the DSMI interface remains intact. (B) Failure strain of different interfaces. (C) Interfacial toughness of different interfaces. (D) Shear strength of different interfaces. (E) Conductivity of electrodes with different connections was measured using the two‐point method. (F) Resistance change of electrodes with SP and DSMI connections under tensile strain. (G) Resistance change of SP‐connected electrodes during repeated stretching between 0%–5%. (H) Resistance at 70% strain during 1000 cycles of 0%–70% cyclic stretching for DSMI‐connected electrodes. (I) Interfacial toughness of various soft‐rigid connections. LM/PDMS/PEIE refers to a composite of liquid metal, polydimethylsiloxane (PDMS), and polyethylenimine ethoxylated (PEIE) [17]; LM/Ag/Epoxy refers to a composite of liquid metal, silver flake, and epoxy resin [18]; LM/SEBS refers to a composite of liquid metal and styrene‐ethylene‐butylene‐styrene (SEBS) [19]; LM/UPy refers to a composite of liquid metal and a polymer containing 2‐amino‐4‐hydroxy‐6‐methylpyrimidine (UPy) motifs [20]. Au/SEBS refers to a composite with a surface modified by a thiol click function group [23]. ACF refers to the 3 m product 9703, data is obtained from the product website (https://www.3m.com/3M/en_US/p/d/b10167835/). (J) Electrical stretchability of various soft‐rigid connections. BIND refers to biphasic, nano‐dispersed interface [12]; KIMI refers to key‐lock type mechanical interlocking [21]. Data in panels (B, C, D, and E) are presented as mean ± standard deviation from 3–5 independent measurements.

The DSMI interfaces exhibited exceptional mechanical stability. The soft conductor sustained 400% tensile strain without interfacial failure on the soft‐rigid connection, with rupture ultimately occurring within the bulk elastomer while the interface remained intact (Figure 2A). This represents a 40‐fold enhancement over ACF‐ and SP‐bonded controls, which delaminated at ∼10% strain (Figure 2B). The ACF‐ and SP‐connections failed because the ACF and SP layers separated from the elastomer, which suggests a significant difference in interface stability compared to the DSMI interface.

The adhesion performance of the interface was tested by a peeling test. ACF‐ and SP‐connections both exhibited adhesive failure at the ACF‐ and SP‐soft electrode interfaces, obtaining low interfacial toughness around 10 J m^−2^. In contrast, the DSMI‐connected interface showed a much higher value beyond 700 J m^−2^, representing a 56‐fold increase (Figure 2C). In addition, examinations after peeling showed that the delamination occurred at the SP‐copper interface, while the SP layer remained on the side of the soft electrode (Figure S5).

Lap shear testing revealed substantial strength differences: ACF‐ and SP‐bonded interfaces failed at around 20 kPa via adhesive separation at the soft electrode interfaces, whereas DSMI specimens exceeded 1.6 MPa before fracture occurred within the rigid backing (Figure 2D and Figure S6). The fracture force reached 40 N for DSMI samples, which is about 20 times larger than the fracture force of an unbacked electrode of the same size. This indicated that DSMI interfaces remained intact beyond the functional limits of the electrode material itself.

Under static conditions, the DSMI connection demonstrated similar conductivity to conventional methods. Two‐point probe measurements showed comparable resistance values for SP‐ and DSMI‐bonded interfaces, while the ACF connections exhibited higher resistance (Figure 1E). The high resistance in ACF‐connections may be ascribed to its poor contact with polymeric electrodes. The comparable resistance of DSMI and SP connections suggests the scaffold does not introduce extra contact resistance in this case.

Dynamic testing revealed critical differences due to interface stability. SP‐bonded interfaces showed a 72% resistance increase at 5% strain before complete failure at 10% strain due to interfacial delamination (Figure 2F). In contrast, the DSMI interface maintained conduction until the electrode fracture at 420% strain. A 100% increase in resistance was observed only at 70% strain (Figure S7), which followed a trend similar to the sample with an interface under no external stress, indicating that no interfacial degradation occurred. Cyclic testing further highlighted DSMI's superiority over SP interfaces. SP connections exhibited a >10×increase in resistance after five cycles at 5% strain (Figure 2G), whereas DSMI specimens subjected to tensile strain cycling, or even to cycling with 180° pre‐twist, exhibited minimal resistance drift (Figure 2H and Figure S8). These results demonstrate that conventional SP bonding is suitable only for static applications, while DSMI enables reliable signal transmission under extreme mechanical deformation.

We compared the performance of our DSMI approach with other connection techniques. In PEDOT:PSS‐based electronics, SP is commonly used for electrical contact. While most works focus on the properties of the soft electrode itself, the interfacial performance is usually not reported (Table S1). Therefore, we compared our method with general soft‐rigid conductive connections that have reported interfacial toughness and/or electrical stretchability (Figure 2I,J and Table S2). Many soft‐rigid connection studies focus on chemical design, such as molecular bond exchange or thiol‐ene reactions, to improve interfacial interaction (Table S2). However, they use hydrophobic polymers like SEBS or PDMS and are mostly applied to metal‐based electrodes, making their methodologies difficult to apply to hydrophilic conductive polymer electrodes. Our DSMI approach relies on structural design, enabling a robust connection between PEDOT:PSS‐based electrodes and metallic interconnects. The interfacial toughness of the DSMI interface reached 730 J m^−^^2^, which is the highest among conductive adhesives based on liquid metal and polymer composites (Figure 2I and Table S2). Since many works do not report the exact interfacial toughness, we also used electrical stretchability for comparison. This value represents the maximum working strain of the soft electrode while pulling the rigid counterpart. The value achieved by our DSMI approach is 420%, which is also superior to reported values (Figure 2J). The electrical stretchability of our method equals the elongation at break of the soft electrode and could be further increased by improving the stretching limit of the electrode. In previous work using a mechanical interlocking method, two micro‐structured gold electrodes disconnected at a low strain of 35%, attributed to the key‐lock topology providing insufficient improvement to interfacial stability [21]. Our thread‐hole design shows a much better effect, rendering the interface damage‐free. Consequently, by prioritizing structural design over chemical modification, DSMI delivers high toughness and stretchability, offering a high‐performance solution for soft electronics — particularly hydrophilic PEDOT:PSS‐based devices.

### 2.3 Structure and Mechanism of DSMI Connection

We characterized the interface structure to investigate the bonding mechanisms. In the case of the direct SP connection, there were two interfaces: the soft electrode/SP interface and the SP/copper foil interface. Since the SP contains a hydrophobic resin matrix and the soft electrode is hydrophilic, the adhesion between them is poor. After cutting it with a blade, a clearly visible gap appeared at their interface (Figure S9), indicating that this interface readily separates under external force. This was consistent with the observed poor stability of this connection under tensile stress. In the case of DSMI, additional interfaces were introduced. As the SP is inherently designed to bond metal electrodes and generally possesses high adhesion, this study focused on the interfaces between the CS and the soft electrode, and between the SP and the CS.

**FIGURE 3:**
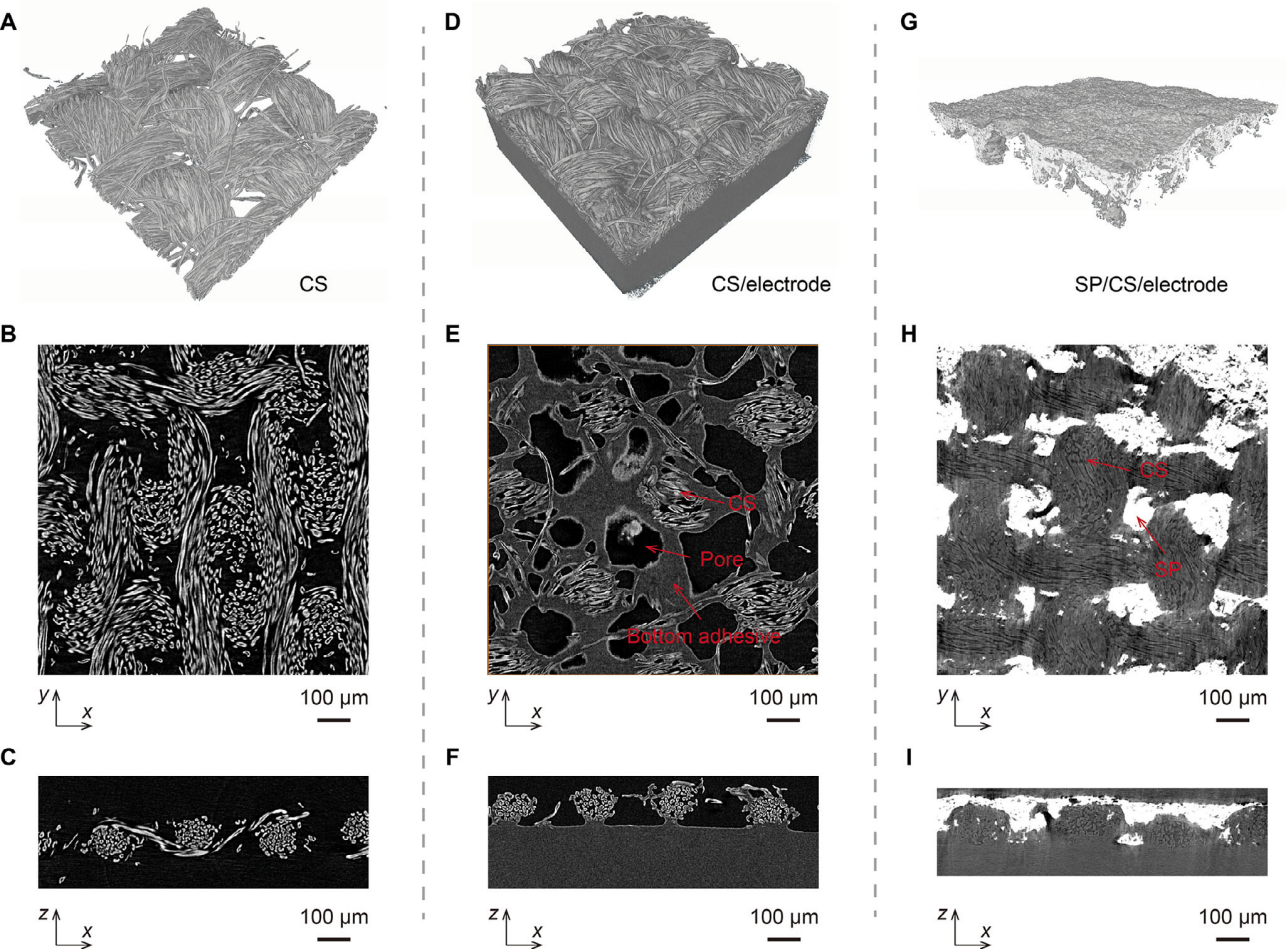
Structure of the DSMI interface. (A) 3D reconstruction of the CS. (B,C) Slides of the CS in the *xy* and *xz* planes, showing the open pore characteristics. (D) 3D reconstruction of the CS/electrode. (E,F) Slides of the CS/electrode in the *xy* and *xz* planes, where the bottom adhesive wraps part of the CS fibers, forming a thread‐hole structure. (G) 3D reconstruction of the SP filled in the CS/electrode. (H,I) Slides of the *xy* and *xz* planes, showing that SP fills the pores of the CS.

The DSMI connection is based on a 60‐count cotton fabric coated with a conductive layer. This scaffold exhibits a hierarchical architecture in which twisted staple fibers form yarns that are further interlaced into a plain‐woven structure (Figure 3A). Sectional images (Figure 3B,C) obtained from micro‐computed tomography (micro‐CT) showed that the fiber‐to‐fiber spacing was approximately 10 µm. The yarns were arranged orthogonally, with a yarn‐to‐yarn spacing of about 100 µm. These fibers and yarns collectively form interconnected open pore channels within the scaffold. The porosity was found to be lowest (∼50%) at the mid‐plane and gradually increased toward the fabric surfaces (Figure S10). This multi‐scale porous structure, featuring both in‐plane and through‐thickness connectivity, offers favorable pathways for adhesive precursor infiltration and enables thread‐hole topological adhesion.

The CS was bonded to the prefabricated soft PEDOT:PSS/WPU electrode using the same precursor solution as an adhesive. Upon drop‐casting, the precursor infiltrated the porous architecture of the CS, and after drying, adhered the scaffold to the electrode surface, as shown in Figure 3D. During infiltration, individual fibers at the bottom became partially embedded within the polymer, causing the adhesive to impregnate the lower portion of the scaffold and wrap numerous filaments; this generated a thread‐hole topological adhesion that mechanically interlocks the scaffold and electrode (Figure 3E,F). Because the precursor and electrode share identical chemical composition, curing yielded a seamless, monolithic interface with no visible boundary (Figure 3F). The porosity of the scaffold base decreases to nearly zero percent after curing, whereas porosity in the upper layers remains comparable to the original state (Figure S10). A small fraction of pores was filled by the polymer precursor, leaving large pores available for subsequent adhesive infiltration.

The upper side of the scaffold can then be further locked with commercial SP, through which a connection is built to a metallic conductor. We investigated the structure of the CS after applying an SP layer. The precursor of SP in its flowable form filled the remaining pores of the scaffold. After curing, the resin occupies the pores, making direct contact with the underlying electrode. The SP layer showed a structure containing a planar base and many pillars (Figure 3G). The sectional image revealed that the pillars of SP filled the pores of the CS (Figure 3H,I). Thus, they formed thread‐hole interlocking on the upper side.

**FIGURE 4:**
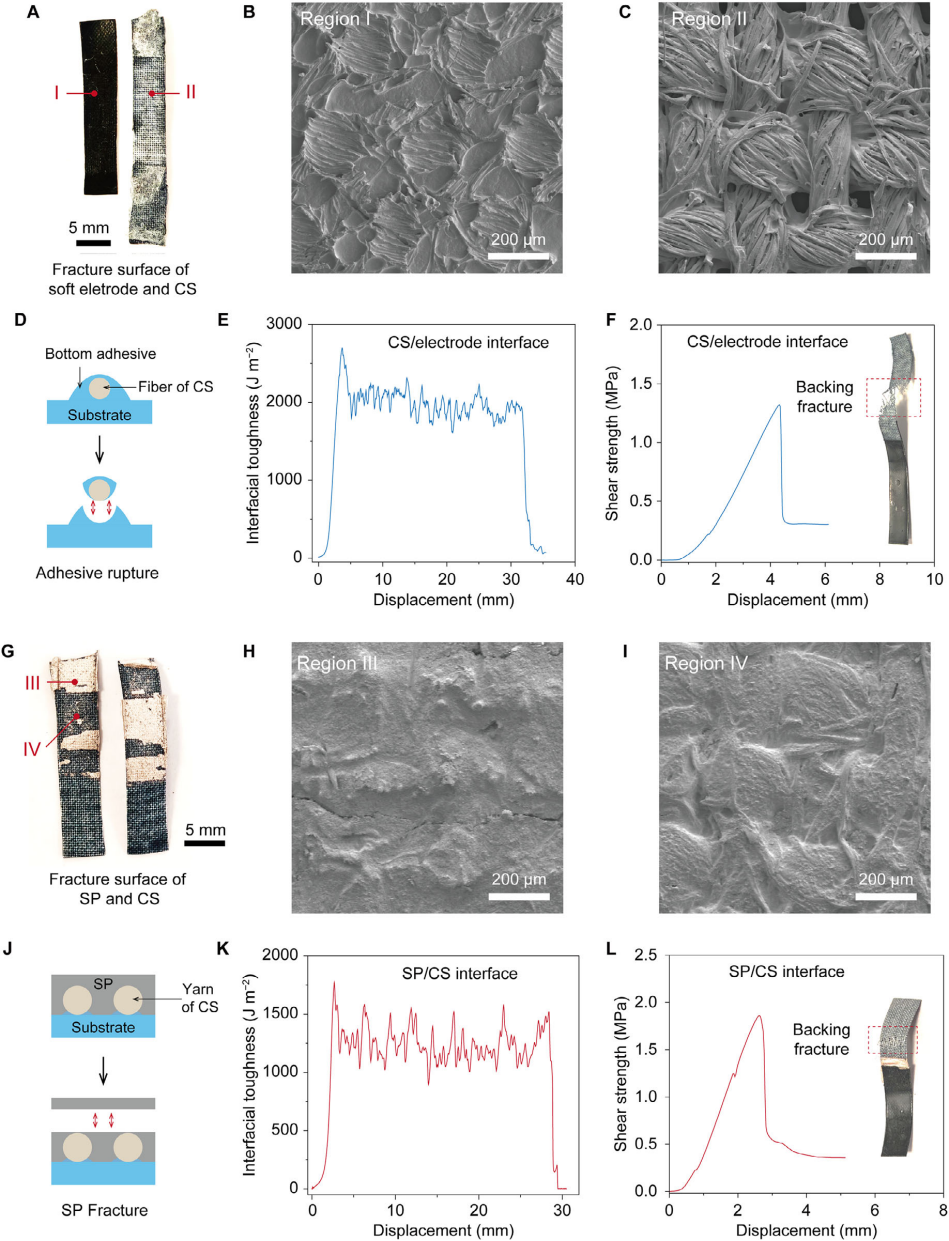
Characterization of DSMI adhesion. (A) Photographs of the CS and electrode after peeling. (B) Surface morphology of the peeled electrode, showing periodic fiber imprints. (C) Surface morphology of the peeled CS, with its surface fibers corresponding to the imprints on B. (D) During the peeling process of the CS and electrode, the adhesive wrapping the CS fibers undergoes bulk fracture. (E) Interface toughness between the CS and electrode during the peeling process. (F) Displacement and stress during the lap shear test of the CS and electrode, with the inset showing that the CS outside the interface fractured while the interface remained intact. (G) Photograph of the SP and CS after peeling, with the silver layer appearing on the surfaces of both samples. (H) The microscopic morphology of region III shows a generally non‐porous surface with fiber imprints. (I) The microscopic morphology of region IV is also non‐porous, with visible fibers filled in. (J) During peeling of the SP and CS, bulk fracture of the SP occurs outside the CS. (K) Interface toughness during peeling of SP and CS. (L) Displacement and stress during lap shear testing of the SP and CS, with the inset showing the fracture of the rigid backing while the interface remains intact.

We peeled the interfaces and observed the fracture surfaces to study how the interface delaminated. Figure 4A shows a photograph of the CS after being peeled from the soft electrode, indicating that the CS completely separated from the soft electrode. SEM observation revealed that the soft electrode surface (Region I) exhibited rough, periodic protrusions and retained striped impressions (Figure 4B). On the opposite side (Region II), the fibers on the CS appeared continuous and intact, without breakage (Figure 4C). Close inspection revealed an excellent morphological match between the fiber pattern in Region II and the impressions in Region I (Figure S11). XPS characterization confirmed the presence of sulfur (S), originating from PEDOT:PSS, on both surfaces (Figure S12). Before peeling, the cross‐sectional CT image (Figure 3E,F) showed that the bottom portion of the CS fibers was embedded in the polymer. Combining this with the post‐peeling observations, it was clear that when the CS is peeled from the soft electrode, the adhesive encapsulating the fibers underwent cohesive failure. One part remained on the soft electrode, while the other part remained within the fiber scaffold (Figure 4D). This failure mode, involving cohesive fracture of the adhesive, entails the breaking of the covalent bonds within the polymer chains, which generally requires more energy than the separation of non‐covalent bonds [30]. As a result, the interface between the CS and the electrode exhibited strong and tough adhesion. In peel testing, the interfacial toughness between the CS and the soft electrode reached 2000 J m^−^^2^ (Figure 4E), a value twice that of the interfacial toughness between cartilage and bone [31, 32]. During shear testing, separation of the CS and soft electrode requires the simultaneous cohesive fracture of the adhesive encapsulating all fibers at the interface. The force required for this is so substantial that in actual testing, the rigid substrate fractured while the interface itself remained intact (Figure 4F).

We then investigated the interface between the SP and the scaffold. Figure 4G shows that the SP was discontinuous and distributed on the surfaces of the two separate samples. SEM observation of two distinct regions, shown in Figure 4H,I, reveals the following: the visibly SP‐covered Region III appeared relatively flat, with shallow fiber impressions. Region IV shows a fully filled surface with no visible pores. While outlines of some fibers remain visible, the spaces between and around them are entirely filled. Additionally, silver (Ag) originating from SP was detected on both surfaces by XPS characterization (Figure S13). Before peeling, the structure of the SP within the CS was shown in Figure 3G–I; its 3D reconstruction revealed a top platform layer and bottom pillars inserted between adjacent yarns. Comparing this to the post‐peeling state, it was apparent that during peeling, cohesive failure occurred within the SP body between the pillars and the platform, with the pillars remaining embedded in the CS (Figure 4J). This fracture mode similarly required significant energy to break the polymer chains within the resin matrix of the SP. Actual testing showed this interfacial toughness also exceeded 1000 J m^−^^2^ (Figure 4K). During shear testing, separation of the interface required the simultaneous fracture of all pillars. The force required for failure exceeds the tensile strength of the rigid backing, and the corresponding shear strength at this point was 1.6 MPa (Figure 4L). By comparing the structure before and after peeling, it is evident that for both interfaces (CS/soft electrode and SP/CS) the adhesives underwent cohesive failure during separation, resulting in very high interfacial adhesion.

### 2.4 Advantage and Limitation of DSMI Strategy

The DSMI strategy is rooted in mechanical interlocking through structural design rather than specific chemical interactions, which decouples interfacial performance from material‐specific chemistry. A DSMI connection comprises three functional elements: (i) the CS, (ii) the soft electrode integrated with its precursor, and (iii) the metal conductor bonded via the SP. Because adhesion arises from topology rather than surface interactions, each component can be independently varied without redesigning the interfacial architecture, enabling robust electrical and mechanical performance across a wide range of material combinations.

The CS functions as the “thread” in the thread‐hole interlocking architecture and plays a vital role in mediating the soft‐rigid interface. Replacing the previously used 60‐count cotton fabric with either a denser (40‐count) or more open‐weave (80‐count) cotton variant, or with a knitted polyester textile (Figure S14), still yielded interfacial toughness values exceeding 700 J m^−^^2^ in DSMI‐based soft‐rigid connections (Figure S15). Microscopic examination of the delamination interfaces, between the CS and the soft electrode, as well as between the CS and the SP, revealed cohesive failure modes consistent with those observed for the 60‐count cotton scaffold (Figures S16–S19), confirming that thread‐hole adhesion is effective across diverse fibrous architectures.

Meanwhile, the sandwich‐type configuration can be adapted into a coplanar layout to reduce interfacial thickness. As a demonstration, a hydrophobic nonwoven polypropylene fabric was sputter‐coated with a thin gold layer on one surface, and both ends of the coated region were bonded, one to a soft electrode and the other to a copper conductor, using the same adhesion protocol (Figure S20). This example highlights the versatility of the CS in the DSMI approach: a wide range of fibrous materials, regardless of wettability, weave pattern, or chemical composition, can be effectively used to construct robust soft‐rigid electrical interfaces compatible with both sandwich and coplanar architectures.

The DSMI connection is compatible with solution‐cast PEDOT:PSS‐based elastomers, which represent a common class of soft conductors. When waterborne WPU is replaced with ethylene‐vinyl acetate (EVA) or styrene‐butadiene rubber (SBR), the same fabrication procedure yields PEDOT:PSS/EVA and PEDOT:PSS/SBR electrodes that achieve interfacial toughness values as high as 2000 J m^−^^2^ with the CS (Figure S21).

Moreover, hydration of the soft conductor alters its surface properties but does not affect its topological structure with the CS. Consequently, the DSMI approach remains effective even for fully hydrated PEDOT:PSS/WPU, which contains 81 wt.% water, comparable to typical hydrogels and thus representative of a hydrogel‐like system [5]. The DSMI connected hydrogel‐metal interface can sustain large tensile strain (Figure S22), which is difficult to achieve with a conventional SP connection.

The choice of SP is also highly flexible, as demonstrated by successful implementation with multiple commercial formulations without requiring any modification to the interfacial design (Figure S23). Similarly, the metal conductor can be tailored to the application, ranging from flexible copper foil to rigid copper‐clad laminate (Figure S24). Together, these results confirm that the DSMI strategy is compatible with a variety of metal conductors and bonding agents, enabling reliable soft‐rigid interfaces across diverse electronic systems.

The DSMI strategy has two notable limitations. The first concerns the solution‐casting step, which inherently restricts the electrical conductivity of both the soft electrode and the adhesive precursor (Table S3). However, this limitation may be addressed through post‐treatment of the electrode to enhance its conductivity, while simultaneously incorporating silver nanowires into the prepolymer solution (used as the adhesive precursor) to improve the conductivity of the adhesive (Figure S25).

The second limitation is more fundamental: the physical dimension of the CS constrains miniaturization. Stable DSMI connections have been achieved with a CS width as small as 0.5 mm (Figure S26), approximately the width of two yarns, but further reduction leads to configurations where a single yarn cannot form a stable mechanical interlock. In addition, both fabric cutting and DSMI bonding are currently performed manually and are not yet compatible with automated manufacturing processes. Addressing these challenges will require further investigation, for example, by exploring alternative scaffold materials with finer features.

### 2.5 Application for Epidermal Electronics

**FIGURE 5:**
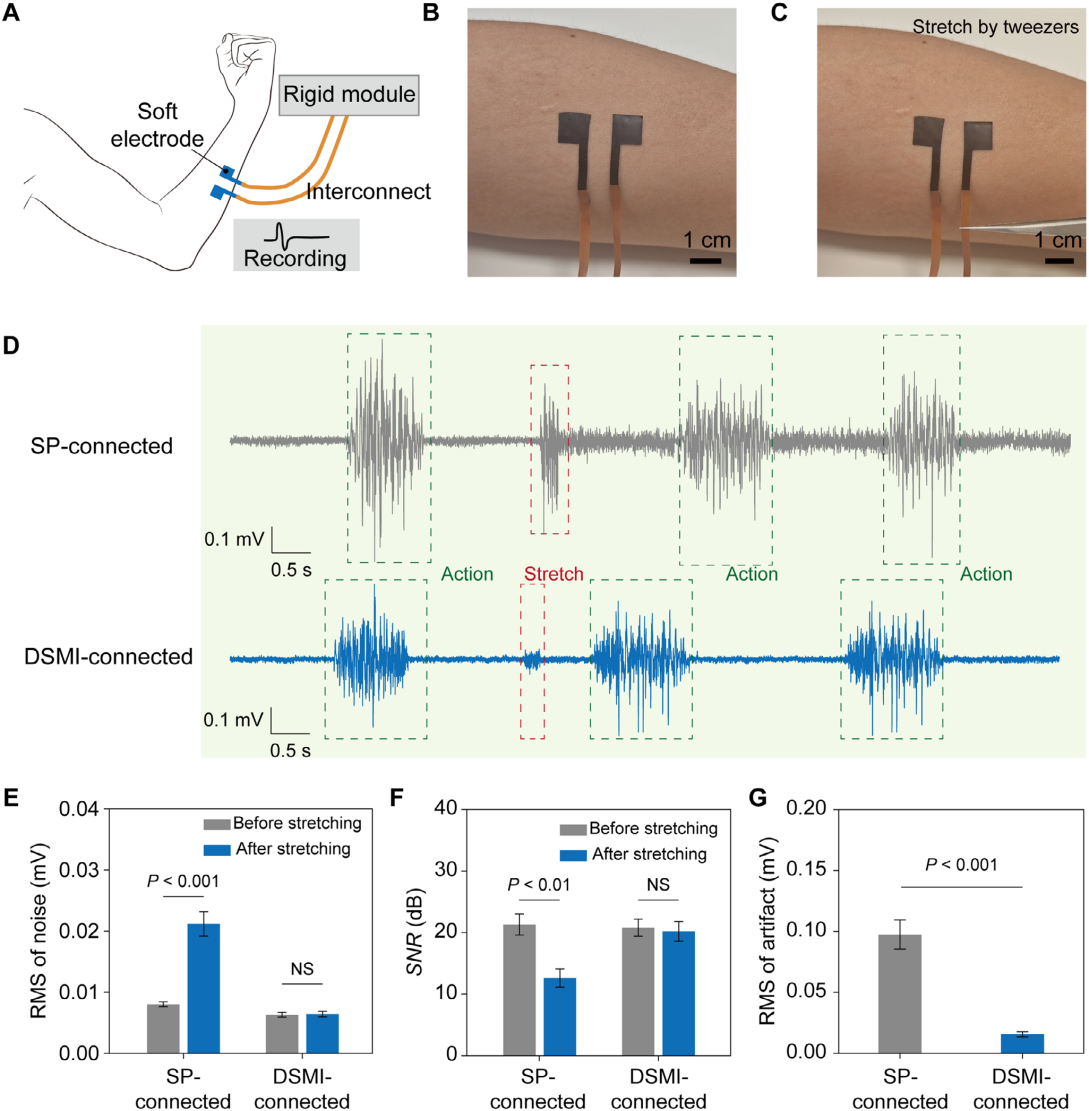
Comparison of epidermal electronics based on SP and DSMI connections. (A) Schematic diagram of EMG signal collection. (B) Photograph of the soft electrode applied to the arm. (C) The soft‐rigid interface is stretched by gently pulling the copper foil with tweezers. (D) EMG signals collected from electrodes with SP and DSMI connections. (E) Noise levels before and after stretching for both electrode groups. (F) Signal‐to‐noise ratio for both electrode groups. (G) Artifacts generated by both electrode groups during stretching. Data in panels (E–G) are presented as mean ± standard deviation from three independent measurements.

Robust and reliable electrical contact is essential for the performance of flexible electronics. As a demonstration, we fabricated EMG recording devices using both SP‐connection and DSMI‐connection for comparison (Figure 5A). PEDOT:PSS/WPU was used as the soft electrode, and an ionic conductive adhesive was applied to enhance contact with the skin [33, 34, 35]. The electrodes were attached to the forearm and connected to copper foil through either SP‐connection or DSMI‐connection (Figure 5B), which was further linked to a recording device. To simulate external disturbance, a stretching force was applied to the soft‐rigid interface by pulling the copper foil with tweezers (Figure 5C). The recorded EMG signals are shown in Figure 5D. After stretching, the SP‐connection showed degraded performance, evidenced by an increased noise level, while the DSMI‐connection remained stable with no visible change. The noise level in the SP‐connection nearly doubled, whereas the DSMI‐connection showed little variation (Figure 5E). As a result, the signal‐to‐noise ratio (*SNR*) of the SP‐connected device dropped to 12.5, approximately a 50% decrease. In contrast, the *SNR* of the DSMI‐connected device remained around 20 even after stretching (Figure 5F). Additionally, the SP‐connection exhibited a much higher level of artifact compared to the DSMI‐connection, with nearly ten times the magnitude (Figure 5G). This demonstration highlights the importance of reliable soft‐rigid electrical interfaces for stable EMG performance under mechanical stress and for minimizing signal artifacts. In this case, the DSMI‐connection clearly exhibits higher interfacial stability than the SP‐connection, maintaining mechanical integrity and consistent electrical performance under deformation. The enhanced interface robustness of DSMI‐connection contributes directly to improved signal fidelity, reduced noise, and minimized artifact, which are critical for high‐quality bioelectronic signal acquisition.

### 2.6 Application for Hydrogel Electrodes

Hydrogel bioelectronics require stable electrical contact between the hydrogel electrodes and the metal interconnects. The presence of water molecules alters surface properties, making it difficult to achieve a stable interface with dry and hydrophobic SP. In contrast, thread‐hole mechanical interlocking is less sensitive to interfacial interactions and preserves the same topological structure even after the polymer electrode swells, thereby providing moderate adhesion under wet conditions.

We evaluated the performance of the DSMI approach in hydrogel bioelectronics. The dry PEDOT:PSS/WPU electrode was bonded to copper via the DSMI interface as described previously. The electrode and its interface were then equilibrated in water to form a hydrogel–metal connection (the fully hydrated PEDOT:PSS/WPU hydrogel has a water content of 81%, comparable to similar systems [5]). A control sample bonded directly via SP was subjected to identical treatment for comparison.

Two‑point measurements showed that the electrical resistance across the DSMI‐connected PEDOT:PSS/WPU hydrogel remained unchanged after hydration (Figure S27a), whereas the interfacial toughness decreased from 700 to ∼300 J m^−^^2^ (Figure S27b), with cohesive failure now occurring at the CS‐electrode interface. This reduction in toughness and shift in failure mode, relative to the dry condition, suggests that water uptake softens the polymer, leading to degraded interfacial stability. Nevertheless, the interface remained intact after three weeks of immersion (Figure S27b). Despite the reduced strength, it could still withstand tensile strains exceeding 300% (Figure S22).

**FIGURE 6:**
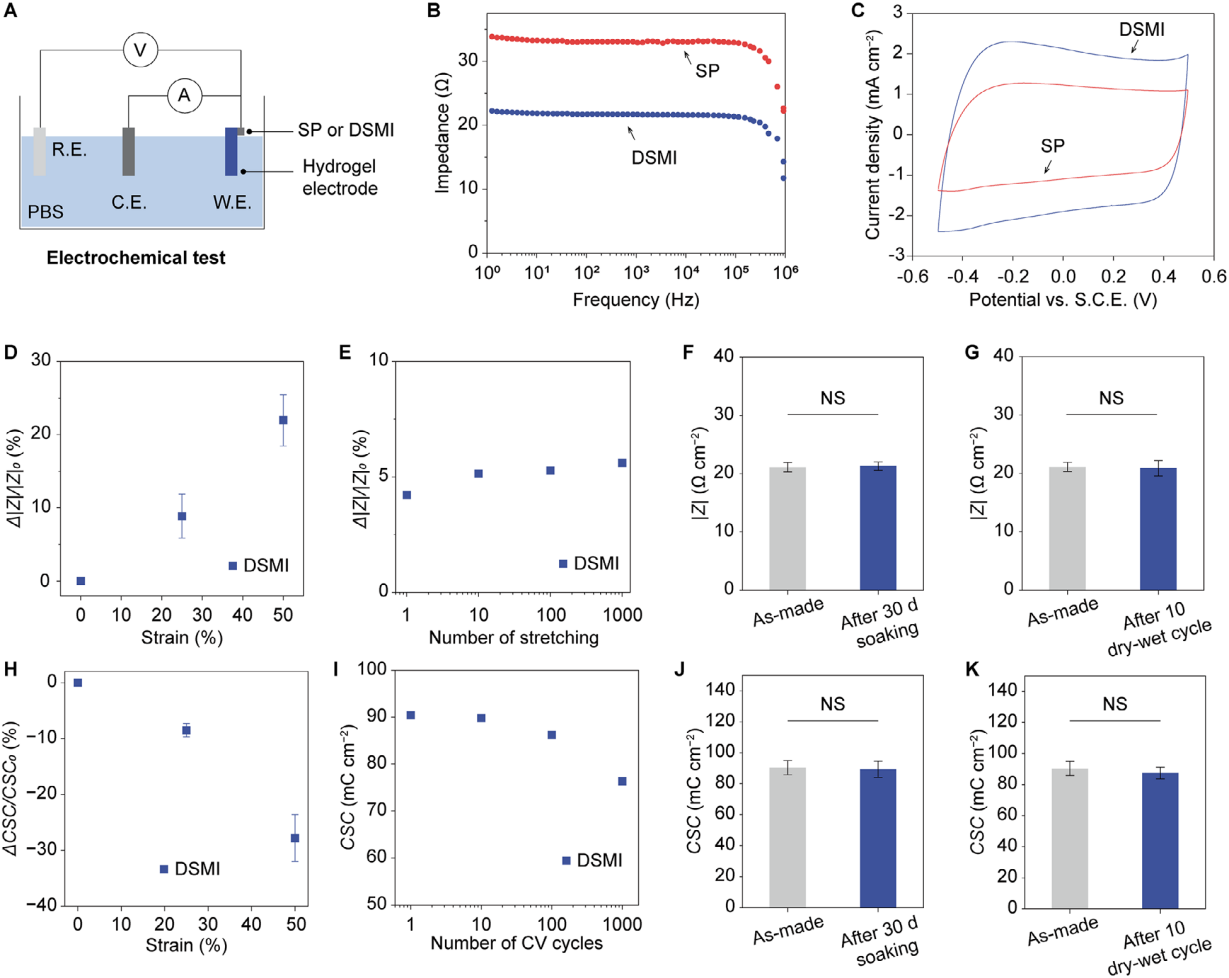
Electrochemical performance of the DSMI‐connected hydrogel electrode. (A) Schematic of the electrochemical testing setup. (B) Impedance spectra of PEDOT:PSS/WPU electrodes connected via DSMI or SP. (C) Current density at different voltage of the hydrogel electrode. (D) Impedance of the DSMI‐connected electrode under tensile strain. (E) Impedance evolution during cyclic stretching. (F) Impedance stability during 30‐day storage in PBS. (G) Impedance response over 10 dry–wet cycling tests. (H) *CSC* of the DSMI‐connected electrode under tensile strain. (I) *CSC* during 1000 cyclic CV cycles. (J) *CSC* stability after 30‐day storage in PBS. (K) CSC response after 10 dry–wet cycling tests. Data in panels (D, F, G, H, J, and K) are presented as mean ± standard deviation from 3–5 independent measurements.

A three‐electrode method is used to compare the electrochemical performance of hydrogel electrodes connected by DSMI and SP (Figure 6A). The PEDOT:PSS/WPU electrode has an area of 1 cm^2,^ while the soft‐rigid interface has dimensions of 5 × 5 × 0.1 mm^3^. Electrodes connected with DSMI exhibited lower impedance (Figure 6B) and greater charge storage capacity (*CSC*) (Figure 6C) compared to those connected with SP. The higher impedance and lower *CSC* with SP likely stem from its weak interface with the hydrogel, which increases contact resistance.

The impedance of the hydrogel electrode was evaluated under mechanical deformation and long‐term environmental exposure. Conventional SP bonding failed under strain due to interfacial delamination and could not be measured. In contrast, the DSMI‐connected electrode exhibited only modest impedance increases of 9% and 22% at 25% and 50% applied strain, respectively (Figure 6D). After 1000 cyclic stretches to 50% strain, the impedance at 1 kHz in the relaxed state increased by just 5.6% (Figure 6E). Following 30 days of storage in PBS, the impedance at 1 kHz showed no statistically significant difference compared to the as‐made state (Figure 6F), and similarly, no significant change was observed after 10 dry–wet cycling tests (Figure 6G).

The *CSC* was also evaluated under similar conditions. As previously observed, SP‐connected hydrogels failed under strain and could not be measured. In contrast, the DSMI‐connected PEDOT:PSS/WPU hydrogel exhibited only a 28% reduction in *CSC* at a high strain of 50% (Figure 6H). After 1000 cyclic voltammetry (CV) cycles, the *CSC* retention remained at approximately 84% (Figure 6I). Moreover, no statistically significant change in *CSC* was observed after either 30 days of storage in PBS (Figure 6J) or 10 dry‐wet cycling tests (Figure 6K).

These results demonstrate the stable electrochemical performance of the DSMI interface under mechanical strain, prolonged storage, and repeated wet‐dry cycling, supporting its potential for reliable soft–rigid connections in hydrogel bioelectronics.

### 2.7 Application for Replaceable Epidermal Electrodes

DSMI‐interface can be further engineered to enable controlled detachment, facilitating replaceable electrodes in epidermal electronics. Electrodes in prolonged skin contact absorb sweat, degrading their adhesion and performance, thus requiring periodic replacement. Clinical practice necessitates disposable electrodes, demanding new epidermal systems to incorporate replaceability. This creates a paradoxical requirement: stable interfacial integrity during operation yet on‐demand disassembly, significantly complicating interface design.

We propose a DSMI‐based solution: After adhering the CS to the electrode surface, only silver paste is applied atop, forming a metallic terminal (Figure S28). This terminal connects to metallic interconnects via ACF. The DSMI‐ACF connected interface can sustain an external strain of 200% and be separated without damage (Figure S29A). The connection can also be able to record EMG signal with comparable quality to that of DSMI and those of commercial Ag/AgCl electrode (Figure S29B,C). In this configuration, epidermal electronics are partitioned into two components: a reusable acquisition/processing module and a disposable electrode connected via DSMI–ACF. After EMG recording, the ACF can be peeled off, the epidermal electrode discarded, and the host system reused, which is analogous to commercial Ag/AgCl electrodes that are single‑use. This approach establishes a pathway for manufacturing disposable, replaceable epidermal electrodes.

## 3 Conclusion

In this work, we present a structural approach for connecting dissimilar PEDOT:PSS‐based soft electrodes with metal‐based rigid interconnects, a crucial step in assembling flexible electronic devices. This connection leverages the open‐pore characteristics of a fabric scaffold, which allows the infiltration of flowable conductive adhesive that forms a thread‐hole structure upon curing. The thread‐hole type mechanical interlocking fails only when the adhesive undergoes bulk failure, which requires significant energy and contributes to high interfacial toughness. The stable soft‐rigid interface enables the electrode to maintain its functionality under external strain, as demonstrated by the high signal quality even after stretching. The DSMI connection can be used to link hydrogel electrodes as well as to fabricate replaceable electrodes, offering broader applications in various flexible electronics. Our approach is based on structural design and does not rely on the development of new adhesives, providing a versatile and general technique for assembling flexible electronics with different conductors.

## 4 Experimental Section

### 4.1 Materials

The PEDOT:PSS colloidal solution used was the Clevios PH 1000 grade, obtained from Heraeus Epurio (Germany). Ethylene glycol (EG) was obtained from Sigma‐Aldrich. Waterborne polyurethane (WPU) was purchased from Anhui Anda Huatai New Materials Company Limited. The 60‐count cotton and the anisotropic conductive film (ACF) 3 M 9703 were bought from Taobao.com. Silver paste (SP) ZB2561 (silicon matrix), ZB2562 (two‐component epoxy matrix), and ZB2565 (one‐component epoxy matrix) were purchased from Nanjing Zhongbei Technology Company. SP in the text refers to ZB2562 if no product code is provided. Choline chloride (ChCl), acrylic acid (AAc), poly(ethylene glycol) diacrylate (PEGDA, Mw. 600), and 2‐oxoglutaric acid were purchased from Aladdin (Shanghai, China). Other reagents were purchased in analytical grade, and all reagents were used as received. Distilled water was used in the experiments.

### 4.2 Fabrication of Conductive Scaffold (CS)

The 60‐count cotton was first treated with oxygen plasma to improve hydrophilicity, followed by dip‐coating in a mixture composed of 9.5 g PH 1000 and 0.5 g EG for 100 s. The cotton was air‐dried for 2 h and then dried on a hot plate at 100°C for 10 min. The cotton was dip‐coated twice to obtain the CS.

### 4.3 Fabrication of Soft Electrode of PEDOT:PSS/WPU

A mixture containing 10 g of PH 1000 solution, 2.7 g of WPU emulsion (solid content 40 wt.%), and 0.5 g of EG was mixed in a planetary mixer, followed by casting onto a glass plate. The mixture was dried at 60°C for 24 h to obtain the PEDOT:PSS/WPU‐based soft electrode.

### 4.4 Fabrication of Soft‐Rigid Interface

For DSMI‐connection, the first step is adding 200 microliters of PEDOT:PSS/WPU prepolymer solution to one end of a 5 mm wide PEDOT:PSS/WPU soft electrode. A 5 × 5 mm^2^ CS piece was then placed onto the liquid, and the assembly was allowed to stand at room temperature for 1 h before being transferred to a 60°C hot plate for 30 min of drying. Next, the as‐prepared SP precursor was coated onto both the CS and a copper foil. The copper foil was then placed onto the CS, and a 50 g copper block was positioned at the connection area to fix the interface with pressure. The SP was cured completely at room temperature for 24 h to complete the DSMI connection. For direct ACF‐connection, a 5 × 5 mm^2^ ACF piece was adhered between the PEDOT:PSS/WPU electrode and a copper foil, followed by pressing to enhance adhesion. For SP‐connection, the as‐prepared SP precursor was coated onto both the PEDOT:PSS/WPU electrode and a copper foil. The copper foil was then placed onto the electrode, and a 50 g copper block was positioned at the connection area to fix the interface with pressure. The SP was allowed to cure completely at room temperature for 24 h.

### 4.5 Preparation of Epidermal Electrode and EMG Recording

A PEDOT:PSS/WPU film was immersed in an aqueous solution of initiator (5 wt.% 2‐oxoglutaric acid) for 5 min. The treated PEDOT:PSS/SBR film was then placed on a flat plastic plate, and a precursor of the adhesive containing 2.89 g AAc, 7 g of a mixture of ChCl and EG (ChCl:EG in a 1:2 molar ratio), 0.1 g of 2‐oxoglutaric acid, and 0.01 g of PEGDA was dropped onto the film and covered with another glass slide. The thickness of the adhesive precursor was controlled by a spacer with a thickness of 0.1 mm. Afterward, the mold was placed under 365 nm UV radiation for 3 min to polymerize the adhesive. In this way, an adhesive ionic conductor PAAc‐ChCl‐EG was grafted onto the PEDOT:PSS/WPU. This epidermal electrode was then connected to copper foil using DSMI or SP, and the copper foil was linked to the micro‐4 system (Cambridge Electronic Design Limited) for EMG recording.

### 4.6 Tensile Test

Copper foils were connected to both ends of a 5 mm wide PEDOT:PSS/WPU strip via DSMI‐, ACF‐, or SP‐connections. The copper foils were clamped using fixtures, and tensile testing was performed using a universal testing machine (LE3153, Lishi (Shanghai) Instruments Co., Ltd). The initial gauge length was defined as the length of the soft electrode between the two interfaces. The tests were conducted at a strain rate of 100% min^−^^1^, and the results are presented as nominal strain vs. nominal stress.

### 4.7 Adhesion Test

To avoid the formation of a soft electrode during the T‐peeling or lap‐shear test, a strip of 60‐count cotton was adhered to one side of the electrode as a stiff backing. This process is the same as the first step in DSMI‐connection. Then the electrode with stiff backing was used to adhere to copper foil or CS. For the peeling test, the width of the interface is 5 mm. For the lap‐shear test, the overlap area is 5 × 5 mm^2^. The test was conducted on a universal testing machine (LE3153, Lishi (Shanghai) Instruments Co., Ltd) at a speed of 30 mm min^−^^1^.

### 4.8 Electrical Characterization

The electrical conductivity was calculated as the reciprocal of the product of sheet resistance (measured using the two‐point method with a Keithley 2100) and thickness. The change in resistance of the sample under elongation was determined by measuring the resistance at both ends of the sample (using a Keithley 2100) while subjecting the sample to different levels of strain control using a universal testing machine (LE3153, Lishi (Shanghai) Instruments Co., Ltd).

### 4.9 Electrochemical Property Test

The electrochemical characteristics of hydrogels connected via DSMI or SP were evaluated in PBS using an electrochemical workstation (CS series, CorrTest). In this setup, the conductive hydrogel served as the working electrode and was immersed in PBS, while the DSMI or SP interface was kept outside the solution. A platinum plate (1 cm^2^) was used as the counter electrode, and a saturated calomel electrode (SCE) as the reference. Impedance was measured over 1 MHz to 1 Hz. Charge storage capacity (CSC) was determined by cyclic voltammetry with a potential sweep from −0.5 to 0.5 V at a scan rate of 20 mV s^−^^1^.

### 4.10 Structural Characterization

Samples of CS, CS/electrode, and SP‐coated CS/electrode were subjected to CT testing in a micro‐CT (nanoVoxel3000, Sanying Precision Instruments Co., Ltd.) at a resolution of 0.5 µm. The data were processed using Avizo 3D. Scaffold porosity was quantified as follows: First, the solid phase and pore space were segmented in each 2D cross‐sectional slice (*xy*‐plane) using the “Interactive Thresholding” tool. The thresholded images were then processed with the “Separate Objects” tool to isolate connected pore regions. Finally, the “Label Analysis” tool was applied slice by slice to calculate the plane porosity—defined as the pore area fraction in the *xy*‐plane—at each *z*‐position. This yielded a spatially resolved porosity profile along the *z*‐axis. The fractured surface of the sample was sputtered with a thin layer of Au before being observed in a SEM (SU 8220, Hitachi).

### 4.11 Experiments on Human Subjects

All experiments were conducted with approval from the Institutional Review Board at the Southern University of Science and Technology (Protocol No. 20220178). The experiments were carried out by a human volunteer (an author of this study). Written informed consent was obtained from the volunteer prior to the experiment.

### 4.12 Statistical Analysis

All statistical comparisons were performed using MATLAB 2025b. Parametric tests were applied under the assumption of normal data distribution, although formal normality testing was not carried out. For comparisons involving more than two groups, statistical significance was assessed using one‐way analysis of variance (ANOVA), followed by Tukey's post hoc test for multiple pairwise comparisons. When comparing two independent groups, an unpaired two‐sample Student's *t*‐test was employed. In all cases, differences were considered statistically significant at a threshold of *p* ≤ 0.05.

## Conflicts of Interest

The authors declare no conflicts of interest.

## Supporting information




**Supporting File**: adma73649‐sup‐0001‐SuppMat.docx.

## Data Availability

The data that support the findings of this study are available from the corresponding author upon reasonable request.
