# Supplementary material for: Double‐Sided Mechanical Interlocking Enables Soft‐Rigid Conductive Interfaces With a Record High Toughness for Flexible Electronics
Source: Adv Mater. 2026 Jun 9;38(39):e73649. doi: 10.1002/adma.73649 (PMC13361169; doi:10.1002/adma.73649)
Supplement: Supplementary file 1 — Supporting File: adma73649‐sup‐0001‐SuppMat.docx. [file ADMA-38-e73649-s001.docx]

Supporting Information

Double-Sided Mechanical Interlocking Enables Soft-Rigid Conductive Interfaces with a Record High Toughness for Flexible Electronics

*Gang Li, Minkun Cai, Chunyan Cao, Zengbai Ouyang, Hong Fu*, Lingyu Zhao*, Bingang Xu**

Table S1. Summary of representative works employing silver paste for connecting PEDOT:PSS-based electrodes to metal interconnects, with no interfacial stability data reported.

| Electrode | Interconnect | Electric contact materials | Ref. |
| --- | --- | --- | --- |
| PEDOT:PSS/PR-PEGMA | Metal | Silver paste | [1] |
| PEDOT:PSS/PU | Pt wire | Silver paste | [2] |
| PEDOT:PSS/PAAc | Cu wire | Silver paint | [3] |
| PEDOT:PSS/PVA | Metal | Silver paste | [4] |
| PEDOT:PSS/PVA/ citric acid/β-cyclodextrin | Metal | Silver paste | [5] |

Table S2. Overview of recent studies on conductive adhesives and connection techniques for soft-rigid Interfaces.

| Materials | Adhesion mechanism | Performance ^a^ | Ref. |
| --- | --- | --- | --- |
| EGaIn/PDMS/PEIE | Tacky adhesion owing to low modulus | *G* = 70 J m^−2^  *ε* = not available | [6] |
| EGaIn/Ag/Epoxy | Epoxy adhesion | *G* up to 400 J m^−2^  *ε* = not available | [7] |
| EGaIn/SEBS | Self-adhesion of SEBS | *G* = 320 J m^−2^  *ε* = 240% | [8] |
| EGaIn/PDMS with UPy motif | Molecular bond exchange and recombination | *G* ≈ 120 J m^−2^  *ε* = not available | [9] |
| Au/SEBS | Self-adhesion of SEBS | *G* = not available  *ε* = 200% | [10] |
| Au/SEBS | Interfacial thiol-ene reactions | *G* = 200 J m^−2^  *ε* = 60% | [11] |
| Au pillar/PDMS | Key-lock type mechanically interlocking | *G* = not available  *ε* = 35% | [12] |
| ACF 9703 from 3M | Pressure sensitive adhesive | G = 350 J m^−2^  *ε* = not available | [13] |
| **Conductive porous fabric scaffold** | **Double sized thread-hole interlocking** | ***G* = 730 J m^−2^**  ***ε* = 420%** | **This work** |

^a^: Performance indicators are interfacial toughness (G) and electrical stretchability (*ε*), N/A means not available.

Table S3. Comparison of PEDOT:PSS/WPU with representative PEDOT:PSS-based conductive elastomers.

| Composition | Content of PEDOT:PSS  [wt.%] | Electrical conductivity  [S cm^−1^] | Elongation at break  [%] | Ref. |
| --- | --- | --- | --- | --- |
| PEDOT:PSS/NR  (Solution cast) | 10 | 4.1 | 530 | [14] |
| PEDOT:PSS/NR  (Solution cast +  post treatment) | 10 | 87 | 480 | [14] |
| PEDOT:PSS/PEO | 33.3 | 36 | 36 | [15] |
| PEDOT:PSS/Lycra | 10 | 60 | 360 | [16] |
| PEDOT:PSS/PU/PEG | N/A^a^ | 9.4 | 350 | [17] |
| **PEDOT:PSS/WPU**  **(Solution cast ^b^)** | **10** | **7.9** | **420** | **This work** |
| **PEDOT:PSS/WPU**  **(Solution cast +**  **post treatment ^c^)** | **10** | **67** | **360** |  |

^a^: N/A means not available.

^b^: PEDOT:PSS/WPU electrode in the main text is prepared from solution casting.

^c^: Post-treatment was performed by soaking the solution-cast PEDOT:PSS/WPU film in 99% ethylene glycol (EG) at 60 °C for 30 min, followed by redrying at 60 °C for 4 h. This highly conductive sample is prepared to demonstrated the potential of the DSMI method when using highly conductive adhesive.

**Section 1. Comparison of key–lock and thread–hole mechanical interlocking with soft, deformable components**

We compare the adhesive performance of two distinct mechanical interlock architectures—"key-lock” and “thread-hole”—using a model system composed of a rigid material (representing conductive resins, metals, or cotton fibers) and a soft material (e.g., a compliant elastomer or hydrogel). To simplify the analysis, we assume that the rigid component neither fractures nor deforms during debonding, and that the intrinsic adhesion between the rigid and soft materials is weak. Consequently, in the absence of mechanical interlocking, debonding occurs via adhesive failure at the interface.

Our analysis is based on this baseline adhesive-failure scenario. In the key-lock configuration (illustrated in Figure S1A), the rigid component acts as a “key” while the soft material functions as a “lock”. Due to the deformability of the soft phase, pulling the rigid key out of the soft lock causes localized matrix deformation without inducing bulk damage to the soft material. Thus, despite the presence of geometric interlocking, the system exhibits the same topological constraint and failure behavior as non-interlocked interfaces—namely, adhesive failure. In other words, the introduction of key-lock architecture does not alter the fundamental failure mode. As a result, the effective interfacial toughness remains primarily governed by the intrinsic interfacial interactions between the rigid and soft materials.

In contrast, the thread-hole architecture fully encapsulates the rigid component—which, in this context, takes the form of a fiber—within the soft matrix. Complete separation of the two phases therefore requires bulk fracture of the soft material. Figure S1B illustrates three possible debonding pathways, but in all cases, separation inevitably leads to cohesive failure within the soft matrix. Hence, the thread-hole interlock fundamentally shifts the failure mode from interfacial (adhesive) to bulk (cohesive) failure. Under this regime, the effective interfacial toughness is no longer dictated by interfacial chemistry, but rather by the energy dissipated during bulk fracture—specifically, the area of the newly created fracture surface in the soft matrix during debonding.

In summary, for systems comprising weakly adhering rigid and soft materials, the key-lock interlock preserves the original adhesive-failure mechanism, and interfacial toughness remains sensitive to interfacial interactions. By contrast, the thread-hole interlock transforms the failure mode into cohesive fracture of the soft matrix, making interfacial toughness dependent on the bulk mechanical properties of the soft material rather than on interfacial adhesion.


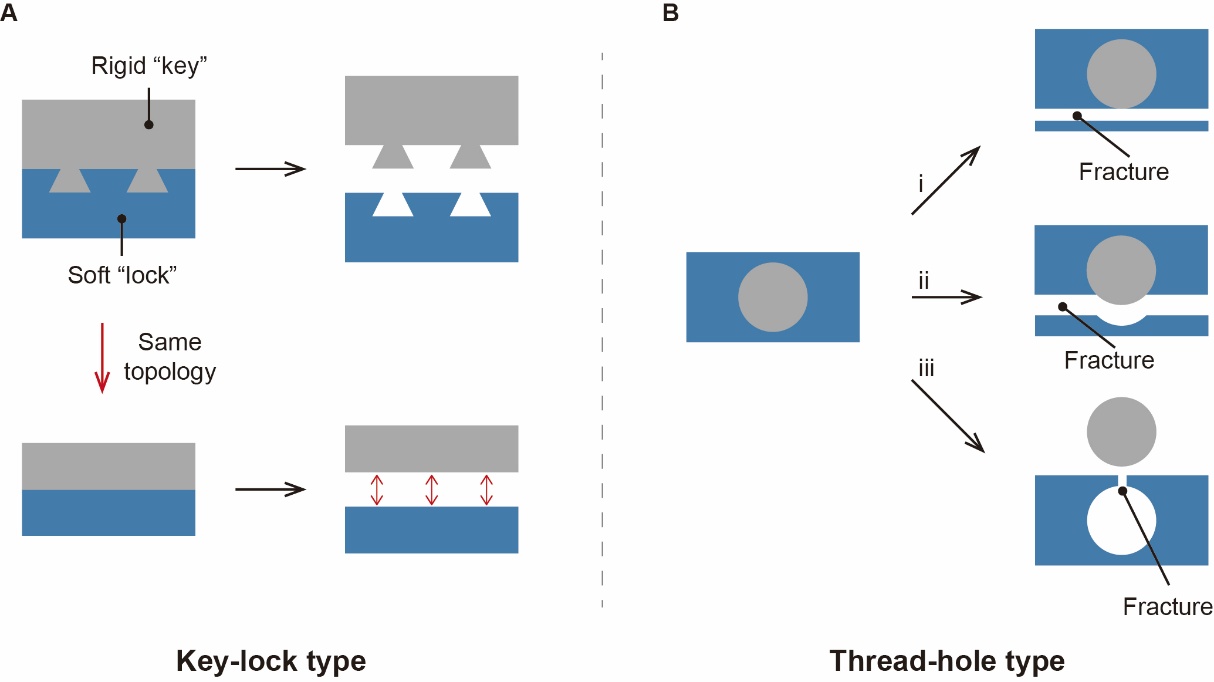


Figure S1. Failure modes of key-lock and thread-hole mechanical interlocking at soft-rigid interfaces. A) In the key-lock design, the deformable nature of the soft matrix prevents effective anchoring of the rigid component, resulting in interfacial (adhesive) failure. B) In the thread-hole architecture, separation of the soft and rigid components consistently induces bulk (cohesive) fracture of the soft matrix.


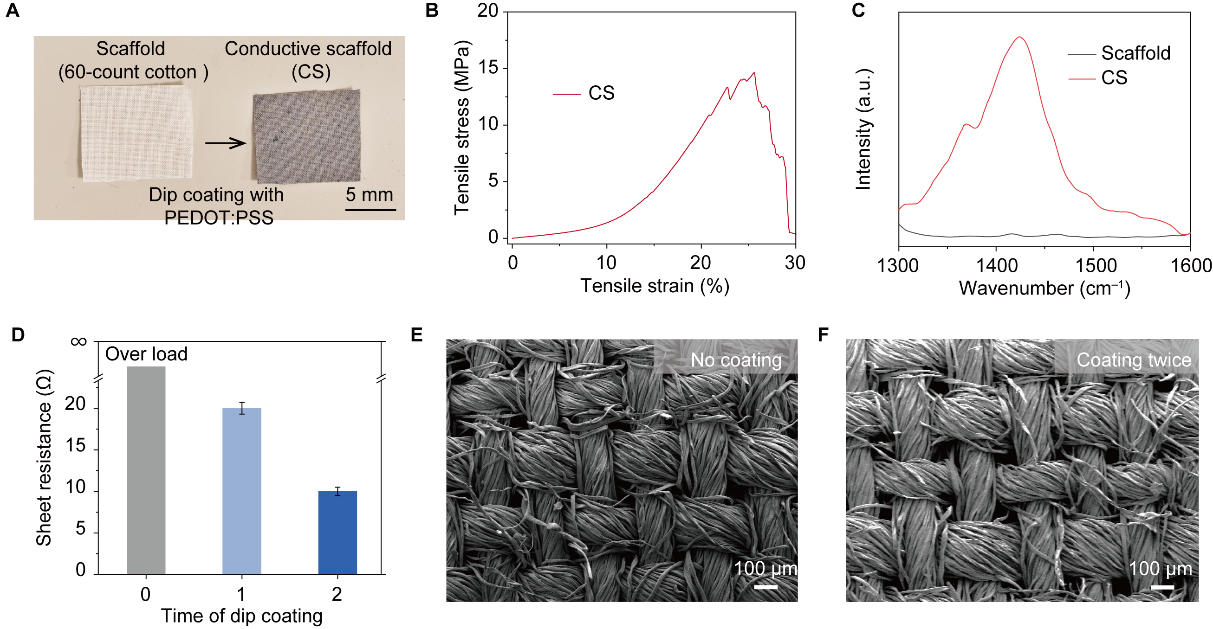


Figure S2. Fabrication of conductive scaffold. A) Photo of the 60-count cotton fabric and the conductive scaffold (CS) after dip-coating the scaffold with PEDOT:PSS twice. B) Tensile strain-stress curve of the CS. C) Raman spectra of scaffold and CS, revealing the characteristic peak of PEDOT present on CS. D) Sheet resistance of the scaffold and the CS with one or two dip coating. E) and F) SEM image of cotton scaffold without and with dip coating. Data in panel D is presented as mean ± standard deviation from 3 independent measurements.


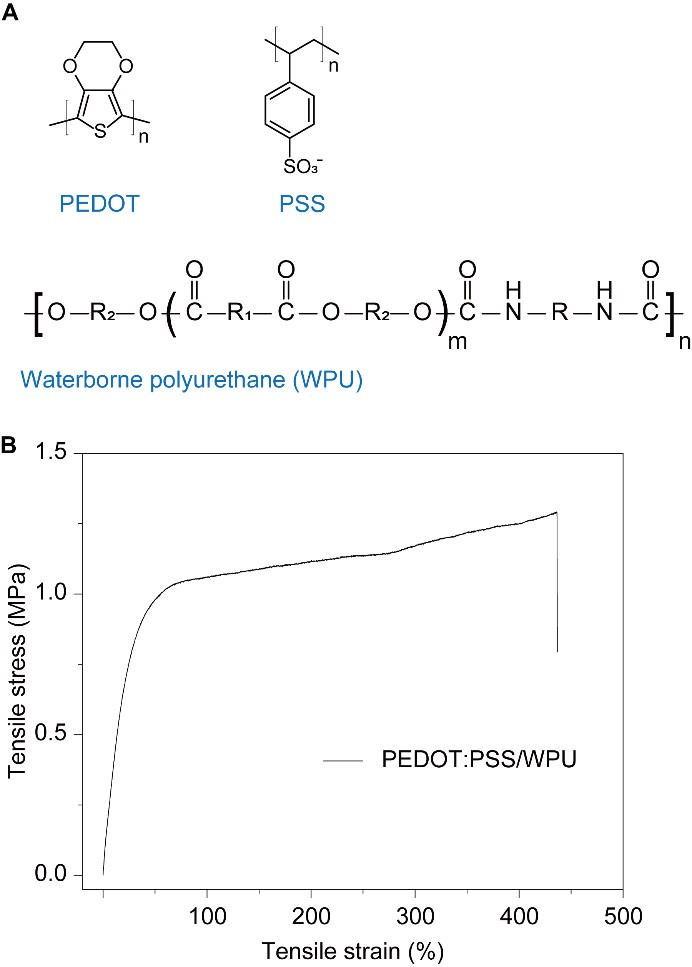


Figure S3. Structure and tensile properties of the soft electrode. A) Molecular structure of PEDOT:PSS and WPU. PEDOT:PSS and WPU blend solution form a conductive elastomer after solution casting. Weight ratio of PEDOT:PSS to WPU is 1:9. B) Tensile strain-stress curve of PEDOT:PSS/WPU soft electrode.


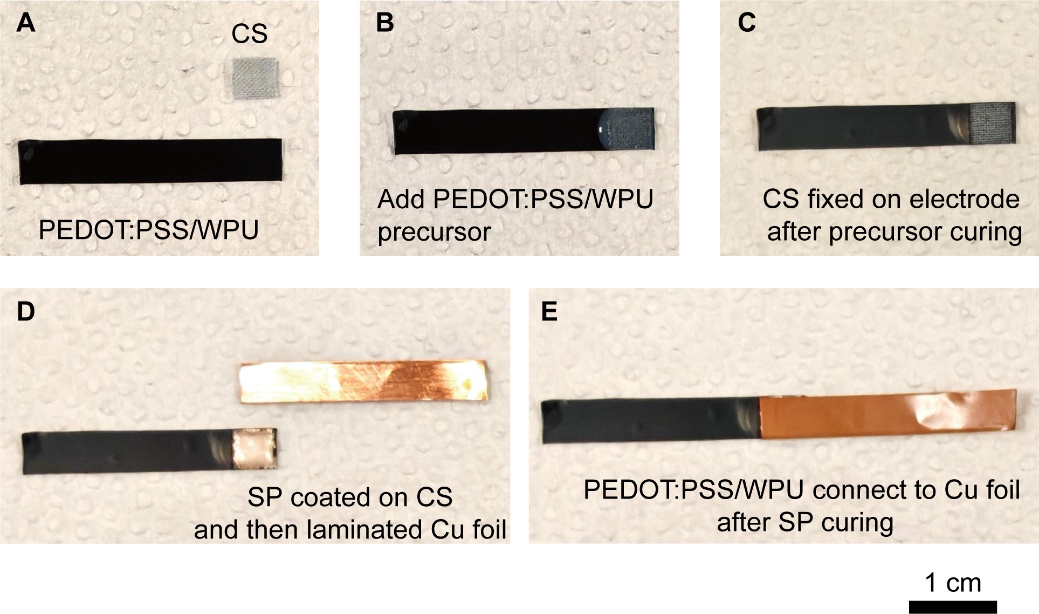


Figure S4. Fabrication of the DSMI connection. A) A piece of CS and a strip of PEDOT:PSS/WPU are prepared. B) PEDOT:PSS/WPU precursor is used as an adhesive and deposited on the surface of elastomer, followed by placing the CS onto the adhesive. C) CS fixed on the electrode after the adhesive is dried. D) SP precursor is coated on the CS, followed by laminated copper foil. E) DSMI connection is built after the curing of SP, which bond both CS and copper foil.


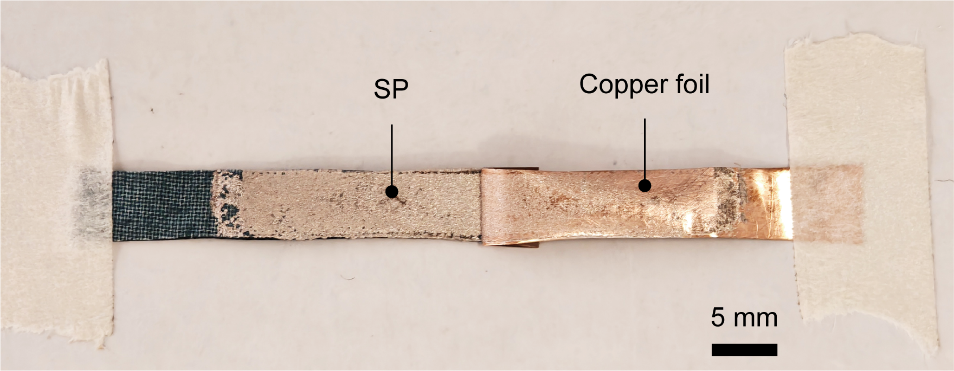


Figure S5. Fracture surface of DSMI-interface after peeling test, which reveals the existence of SP on the CS side and the failure interface is the SP-copper foil.


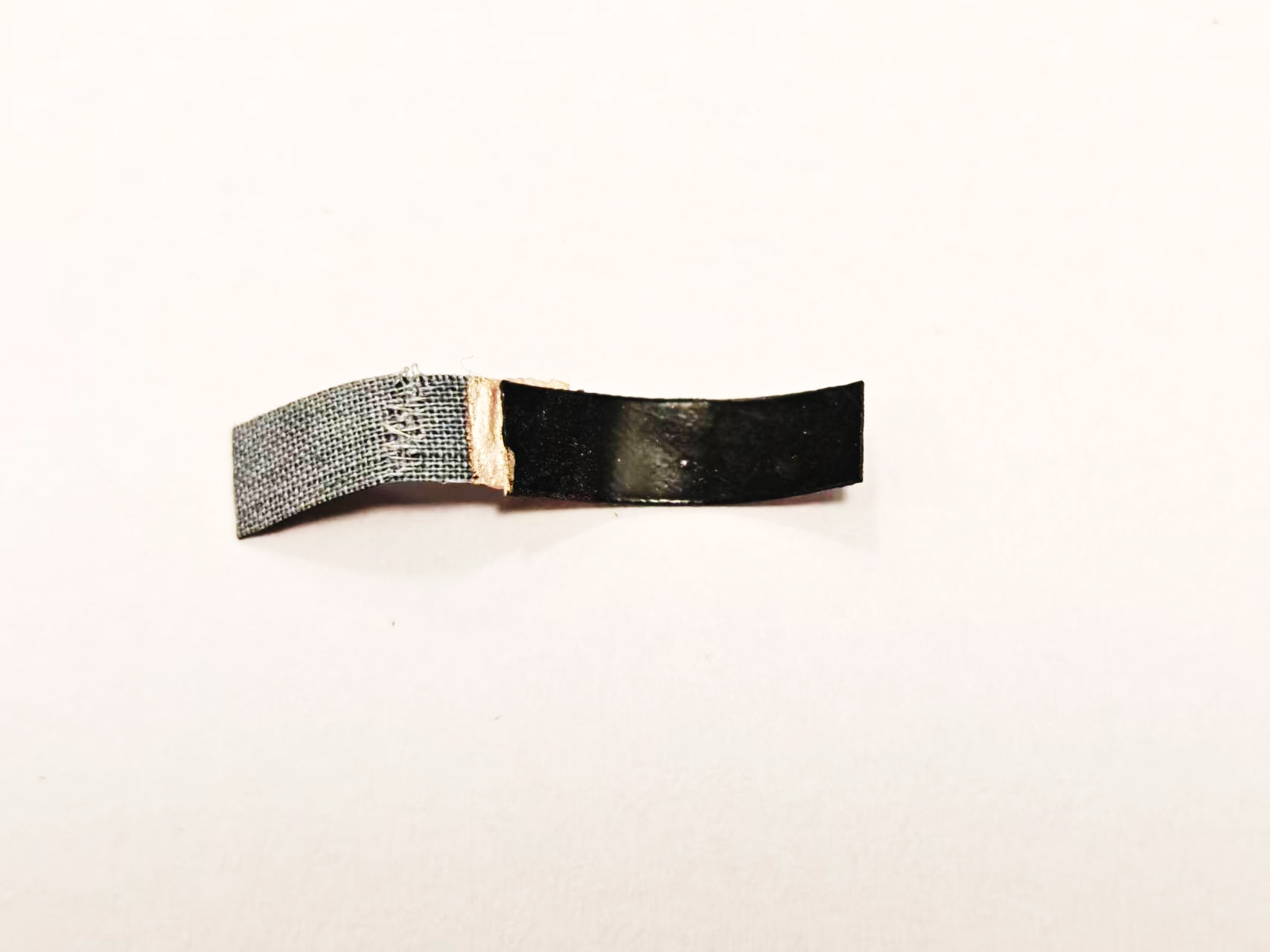


Figure S6. Fracture of stiff backing in lap shear testing, while the DSMI-connected soft-rigid interface remains intact.


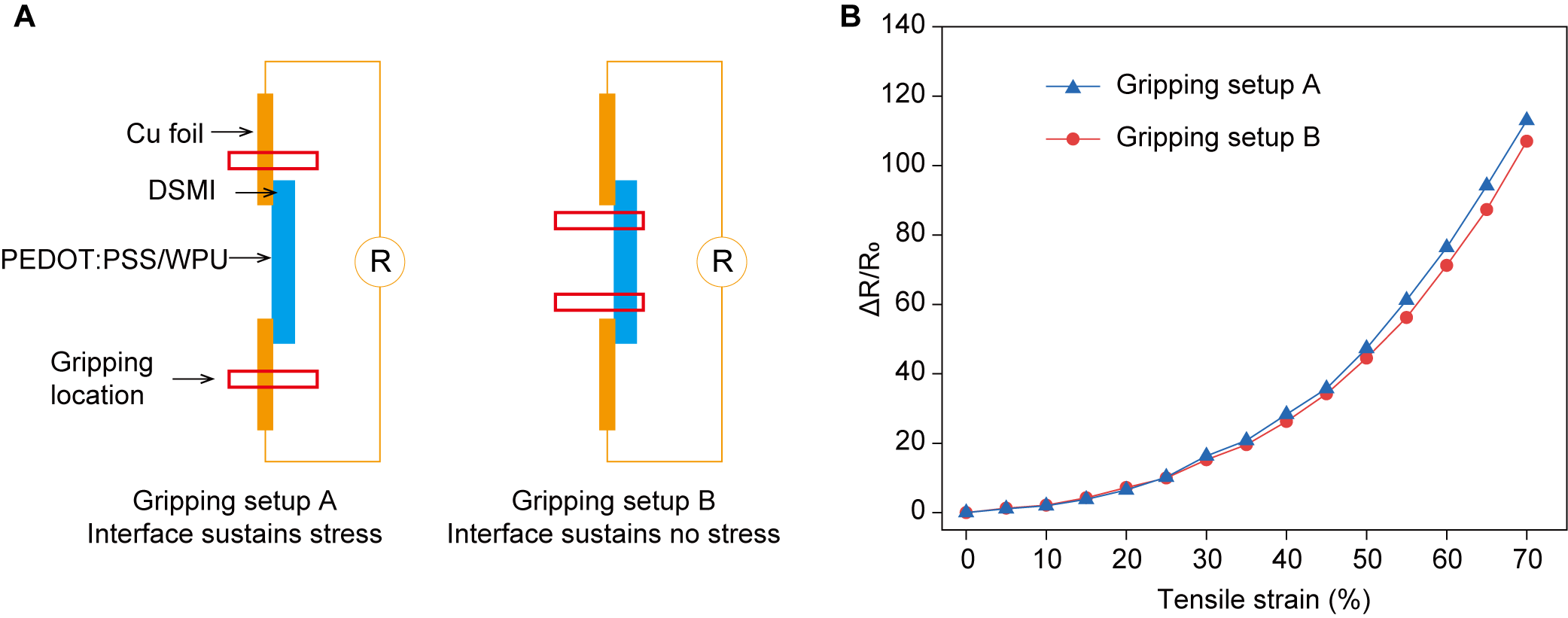


Figure S7. Resistance changes of a soft electrode during stretching of a DSMI-connected sample under different measurement setups. A) Different gripping locations place the DSMI interface either under external stress or free of external stress during stretching. B) Resistance change of the soft electrode for the two gripping setups.


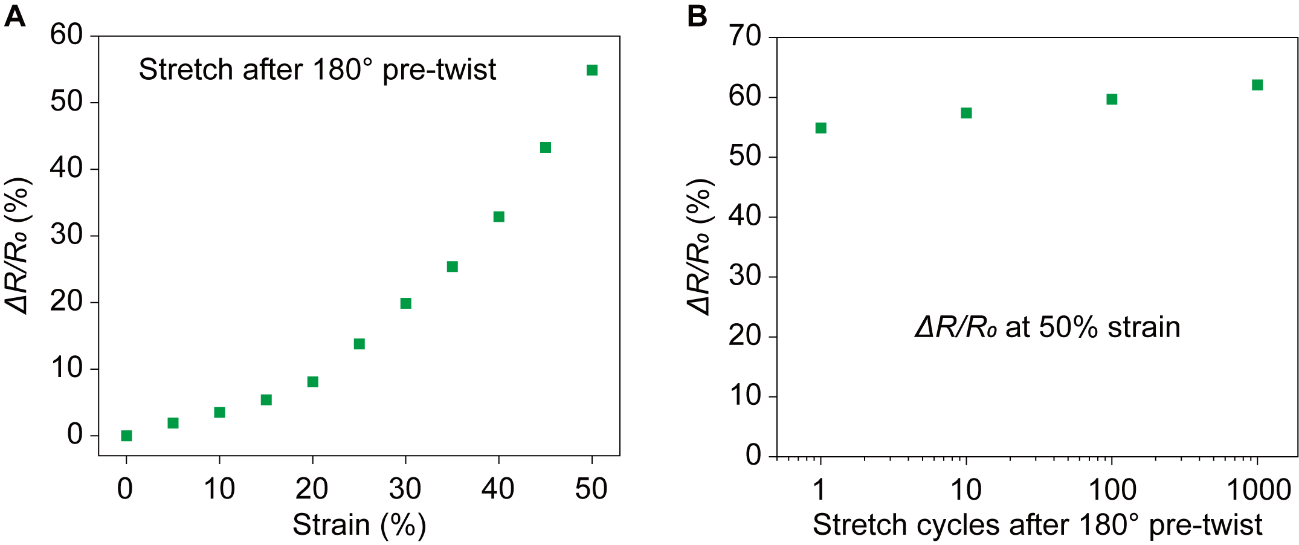


Figure S8. Resistance changes of pre-twisted DSMI-connected soft electrodes during stretching. (A) Change in resistance of the pre-twisted sample at different strains. (B) Change in resistance of the pre-twisted sample during cyclic stretching.


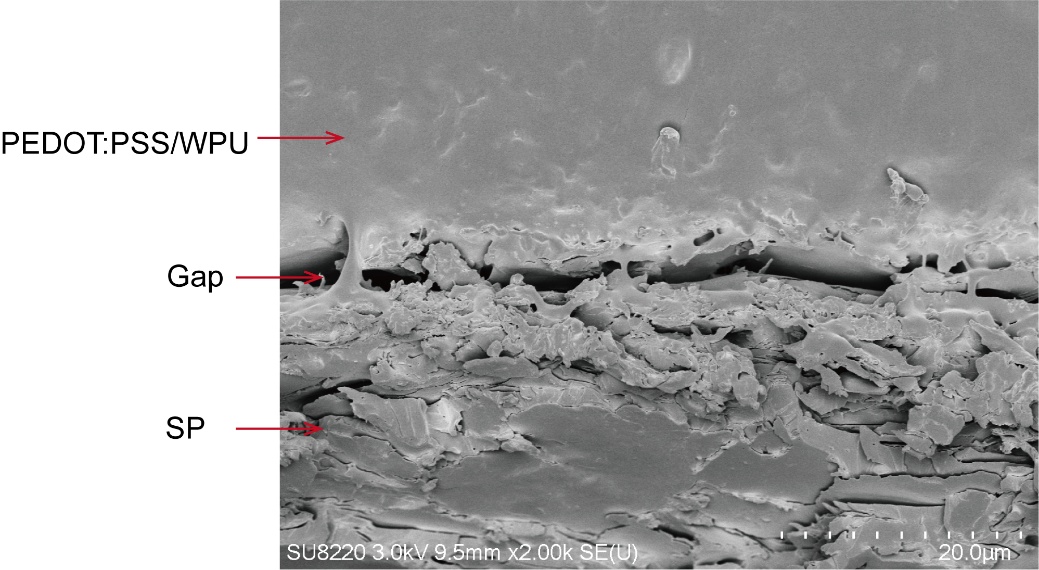


Figure S9. Cross-section of the interface between PEDOT:PSS/WPU and SP. A gap in the order of micrometer is presented between the two layers, indicating the occurrence of delamination.


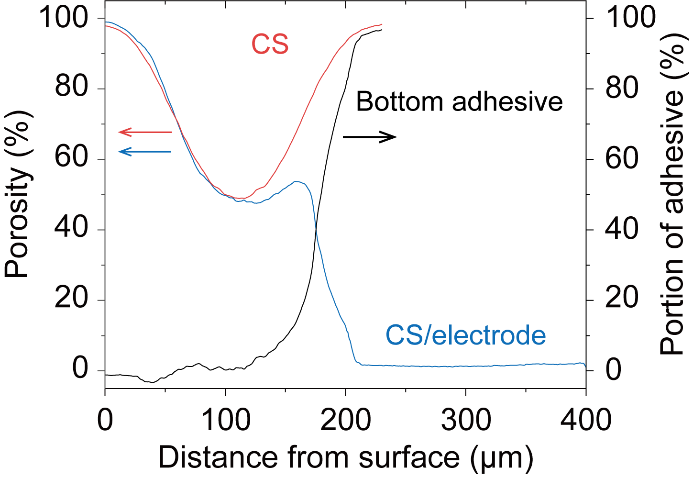


Figure S10. Porosity and filling ratio of bottom adhesive of CS and CS/electrode.


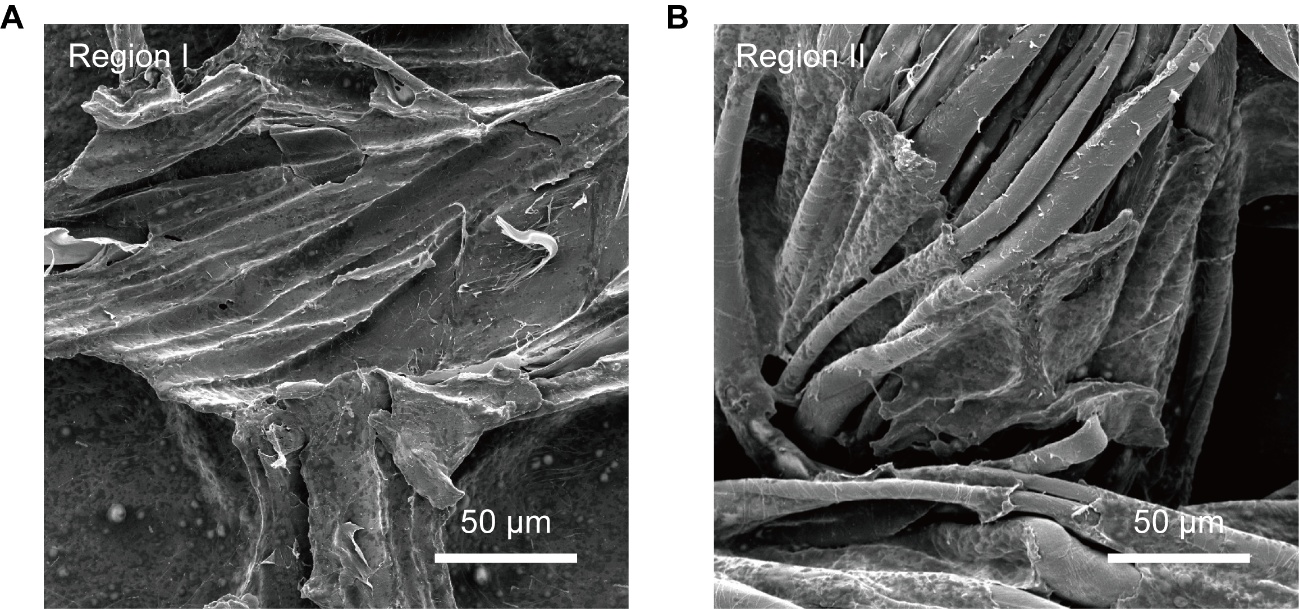


Figure S11. SEM images of the peeled surfaces of the soft electrode and the CS. The surface of the soft electrode reveals an imprint of fibers (A) that matches the shape of the fibers of the CS (B), indicating that the adhesive underwent cohesive failure.


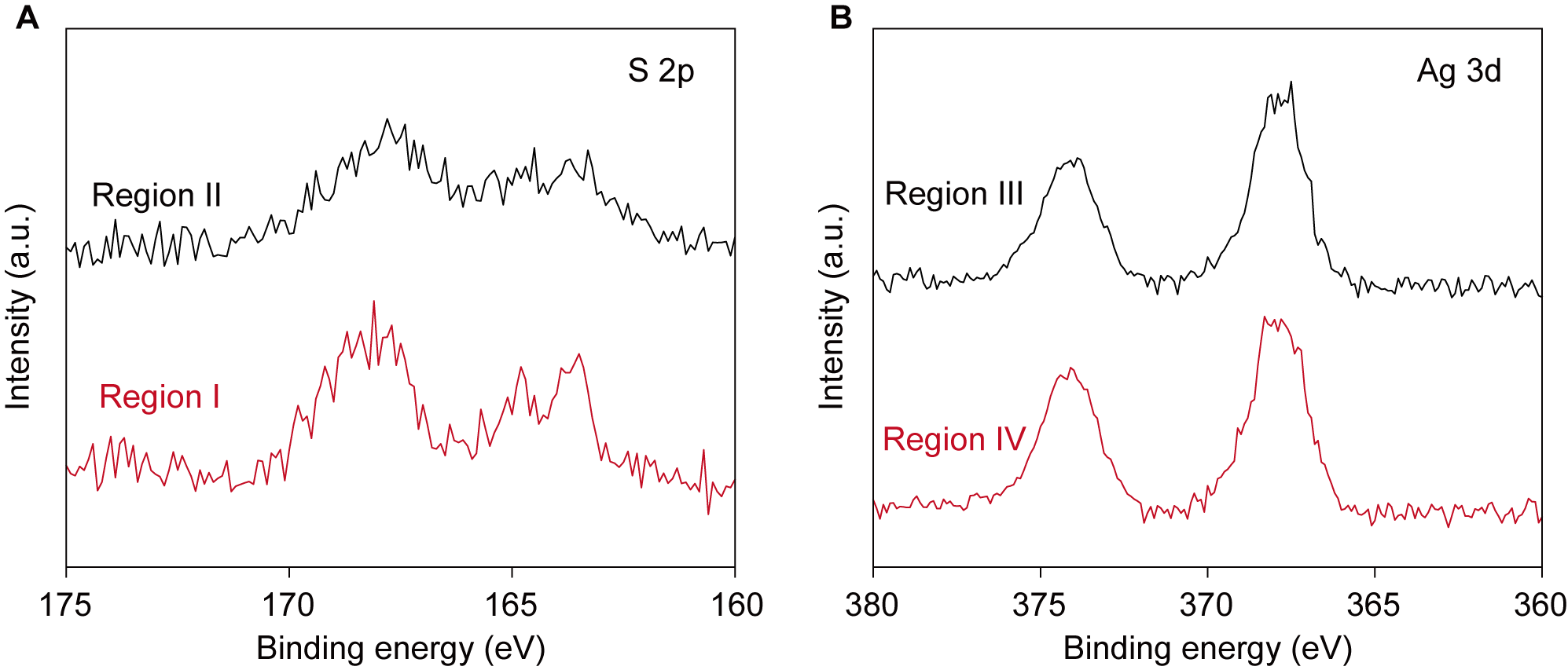


Figure S12. XPS spectra of the peeled surfaces of the soft electrode (Region I) and the CS (Region II). The presence of peaks originating from sulfur (S) atoms in PEDOT and PSS indicates that PEDOT:PSS are present on both surfaces.


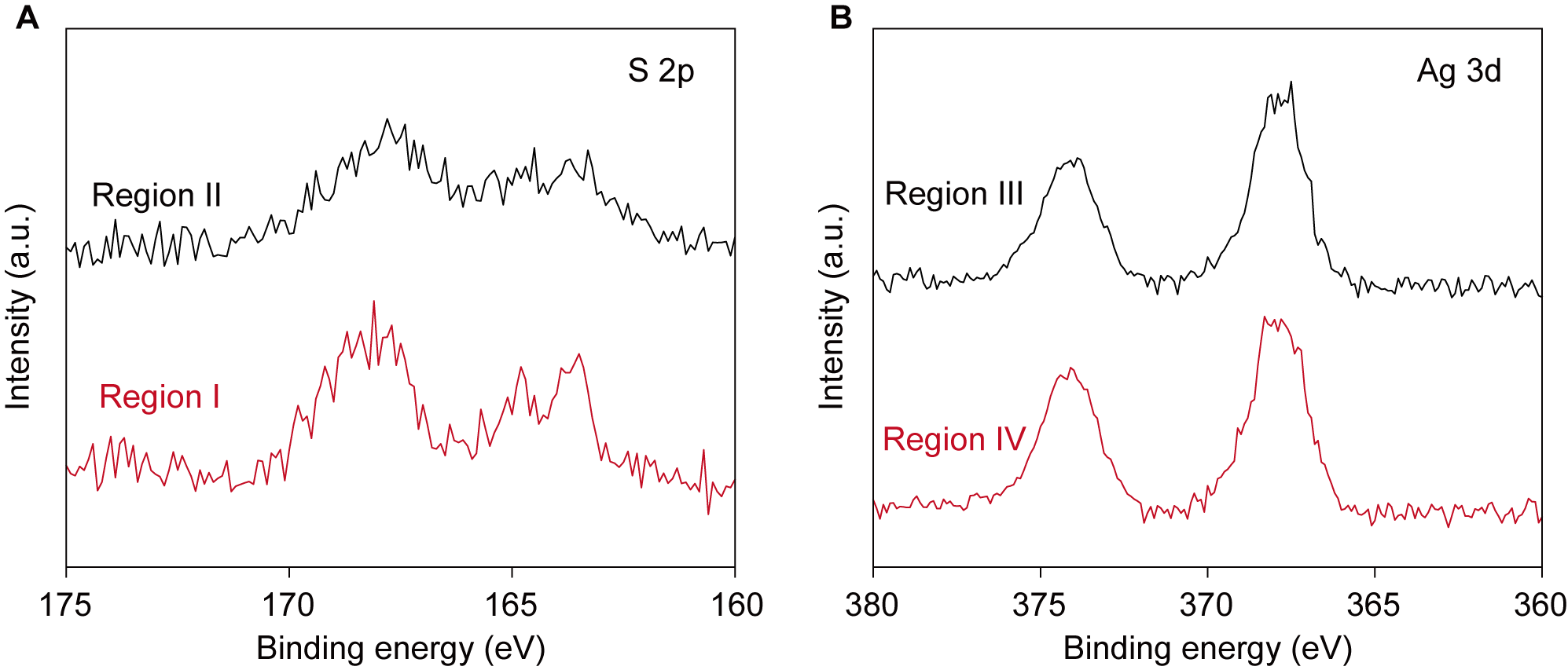


Figure S13. XPS spectra of the peeled surfaces of the SP (Region III) and the CS (Region IV). The detection of characteristic silver peaks confirms the presence of SP on both surfaces.


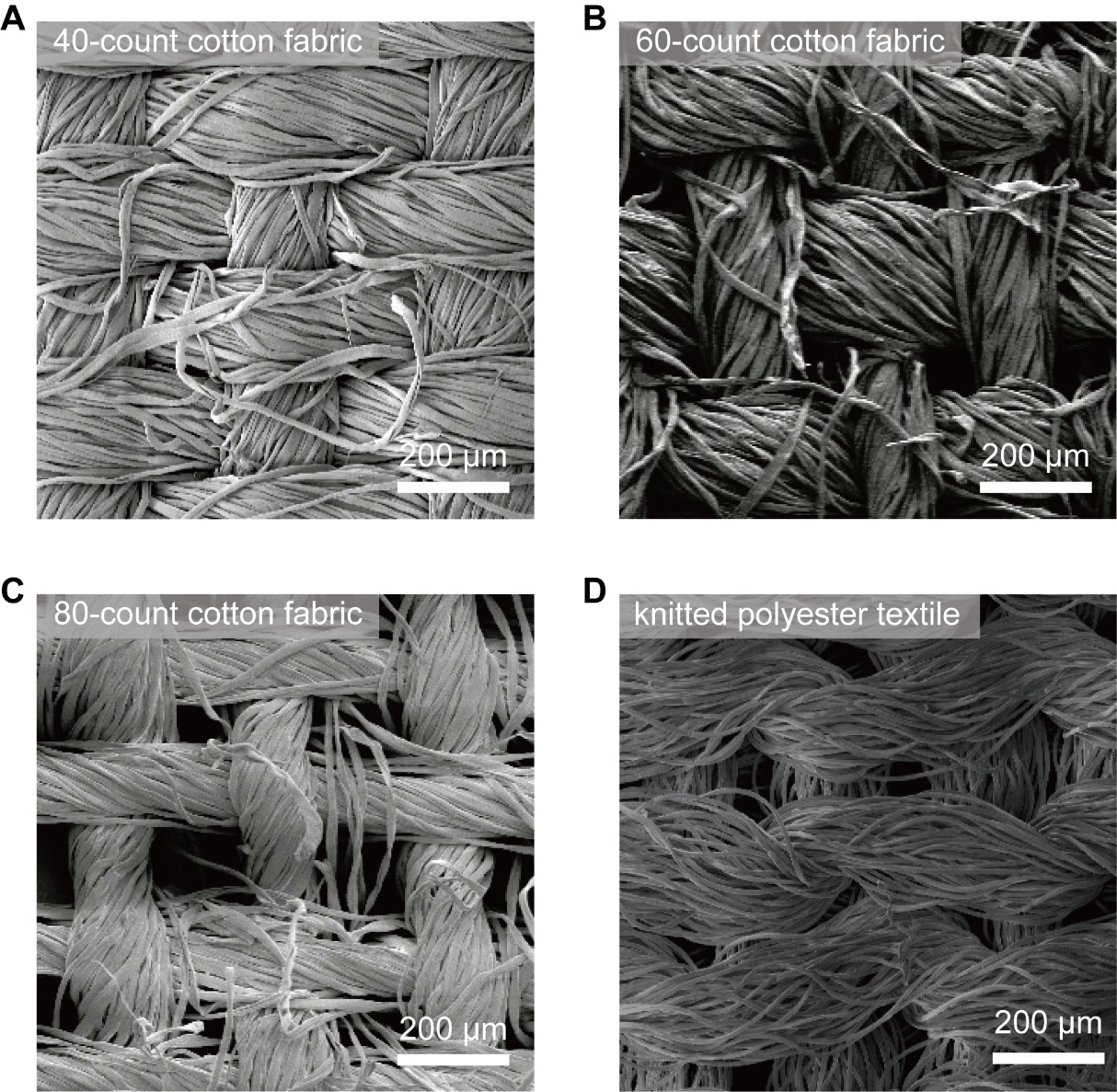


Figure S14. SEM images of various CS. A) 40-count cotton fabric, B) 60-count cotton fabric, C) 80-count cotton fabric, and D) knitted polyester textile.


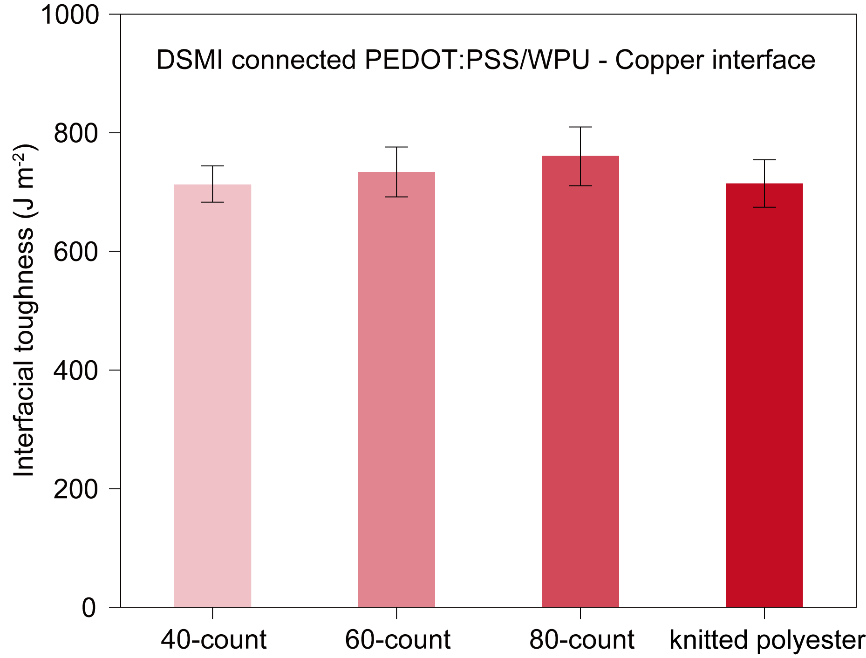


Figure S15. Interfacial toughness of the DSMI interface using CS made from cotton fabrics with different thread counts and a knitted polyester textile. Data are presented as mean ± standard deviation from 3 independent measurements.


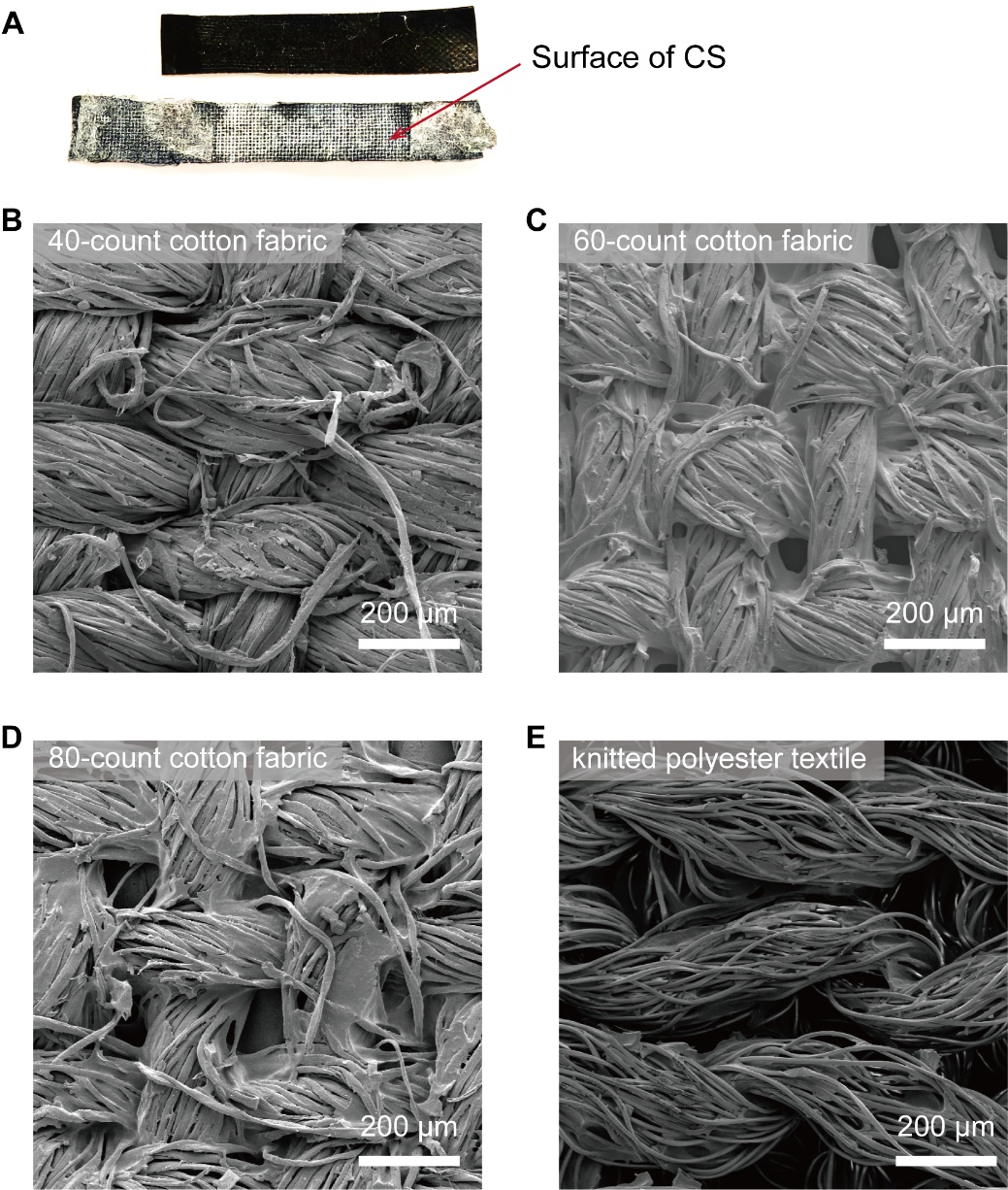


Figure S16. Analysis of the peeled surface of the CS. A) Illustration of the peeled CS surface. B–E) SEM images of (B) 40-count cotton fabric, (C) 60-count cotton fabric, (D) 80-count cotton fabric, and (E) knitted polyester textile. These images reveal a similar morphology, showing that polymer residues appeared inside the yarns.


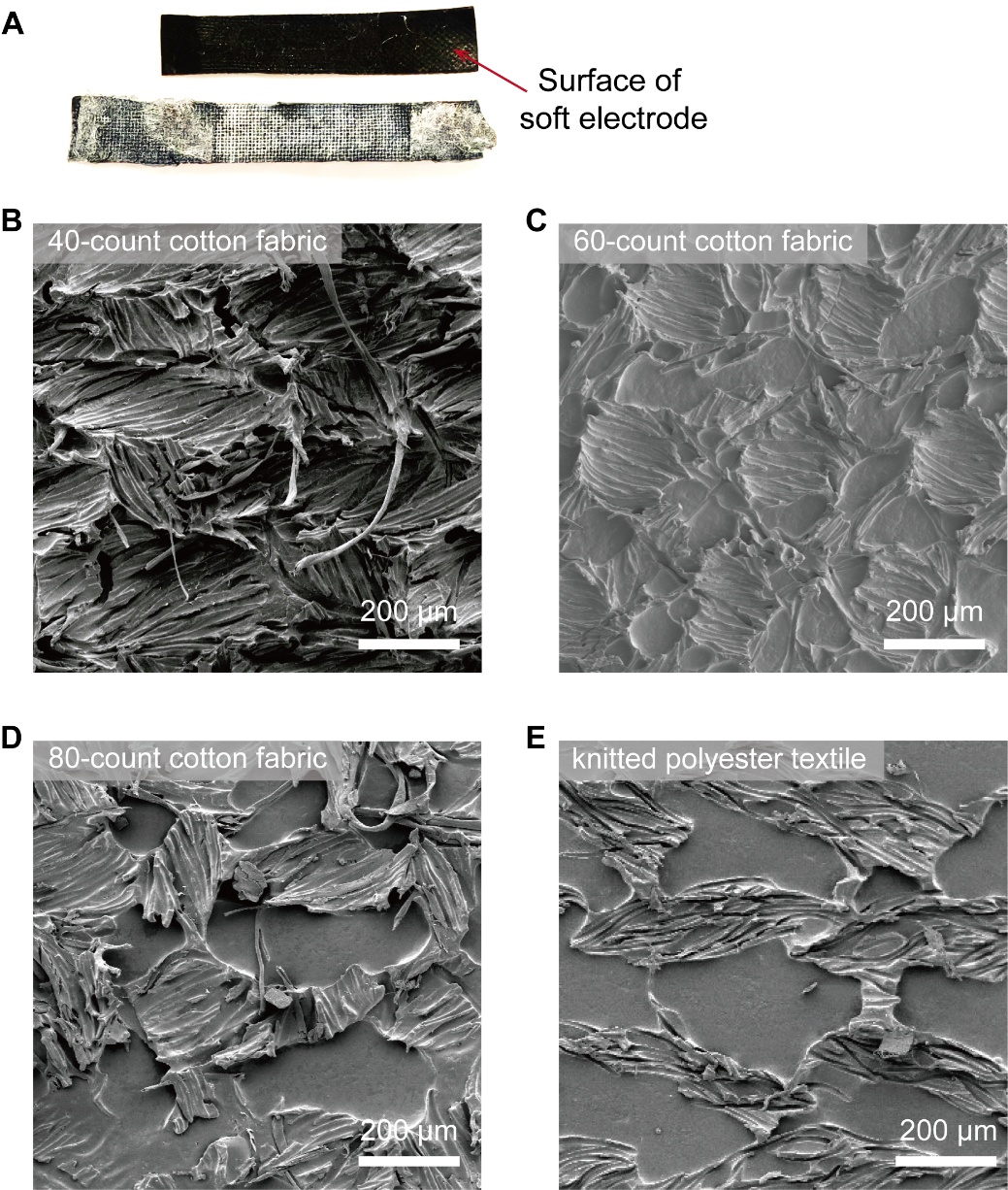


Figure S17. SEM images of the peeled surface of the soft electrode. A) Illustration of the peeled soft electrode surface. B–E) SEM images of soft electrodes peeled from (B) 40-count cotton fabric, (C) 60-count cotton fabric, (D) 80-count cotton fabric, and (E) knitted polyester textile. These images reveal a similar morphology, showing that fabric imprints appeared on the surface of the soft electrodes.


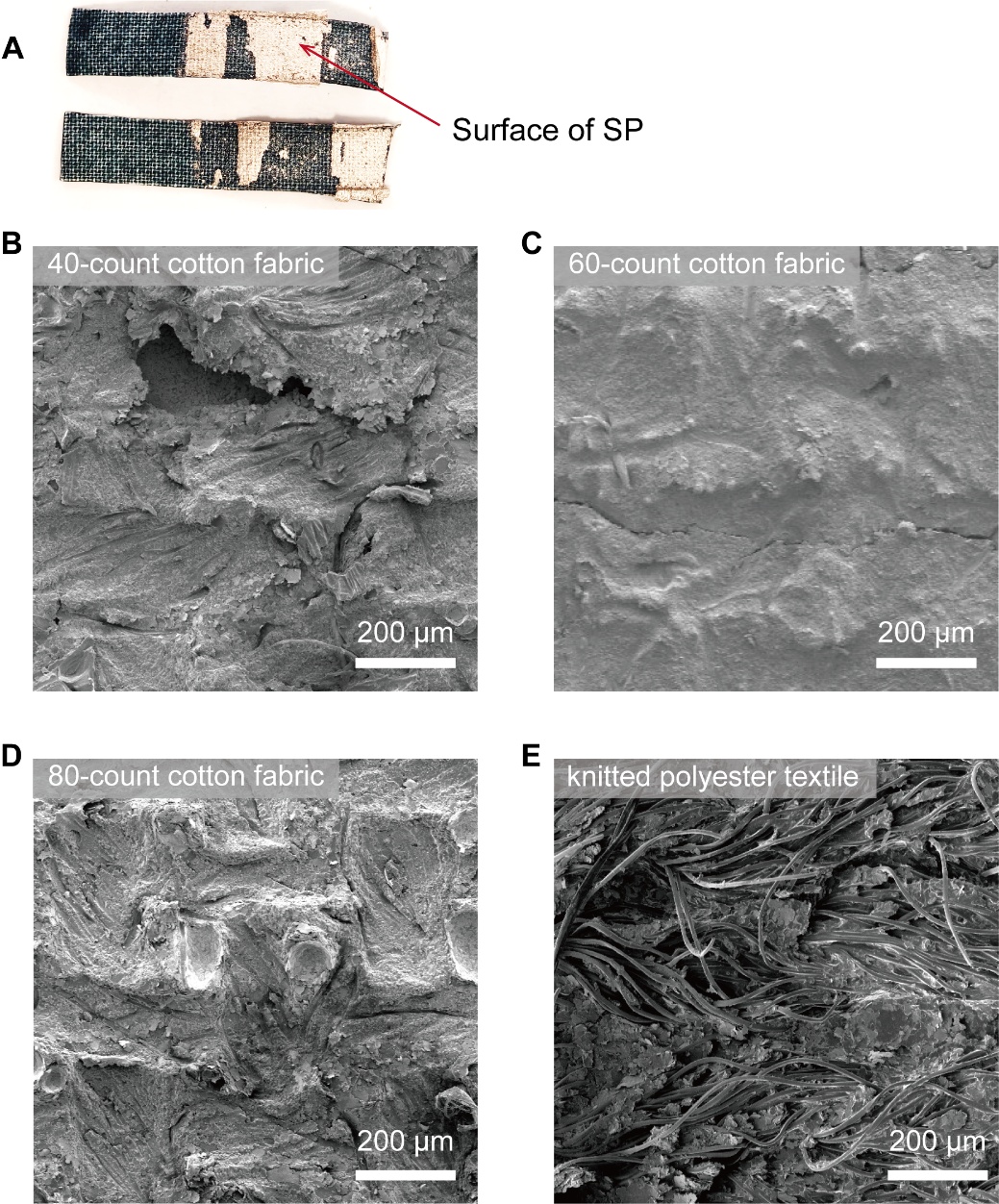


Figure S18. SEM images of the peeled surface of the SP. A) Illustration of the peeled SP surface. B–D) SEM images of SP peeled from (B) 40-count, (C) 60-count, and (D) 80-count cotton fabrics reveal a similar morphology, showing that fabric imprints appeared on the SP surface. In contrast, for the knitted polyester textile-based CS, the SP was found filled inside the fibers (E).


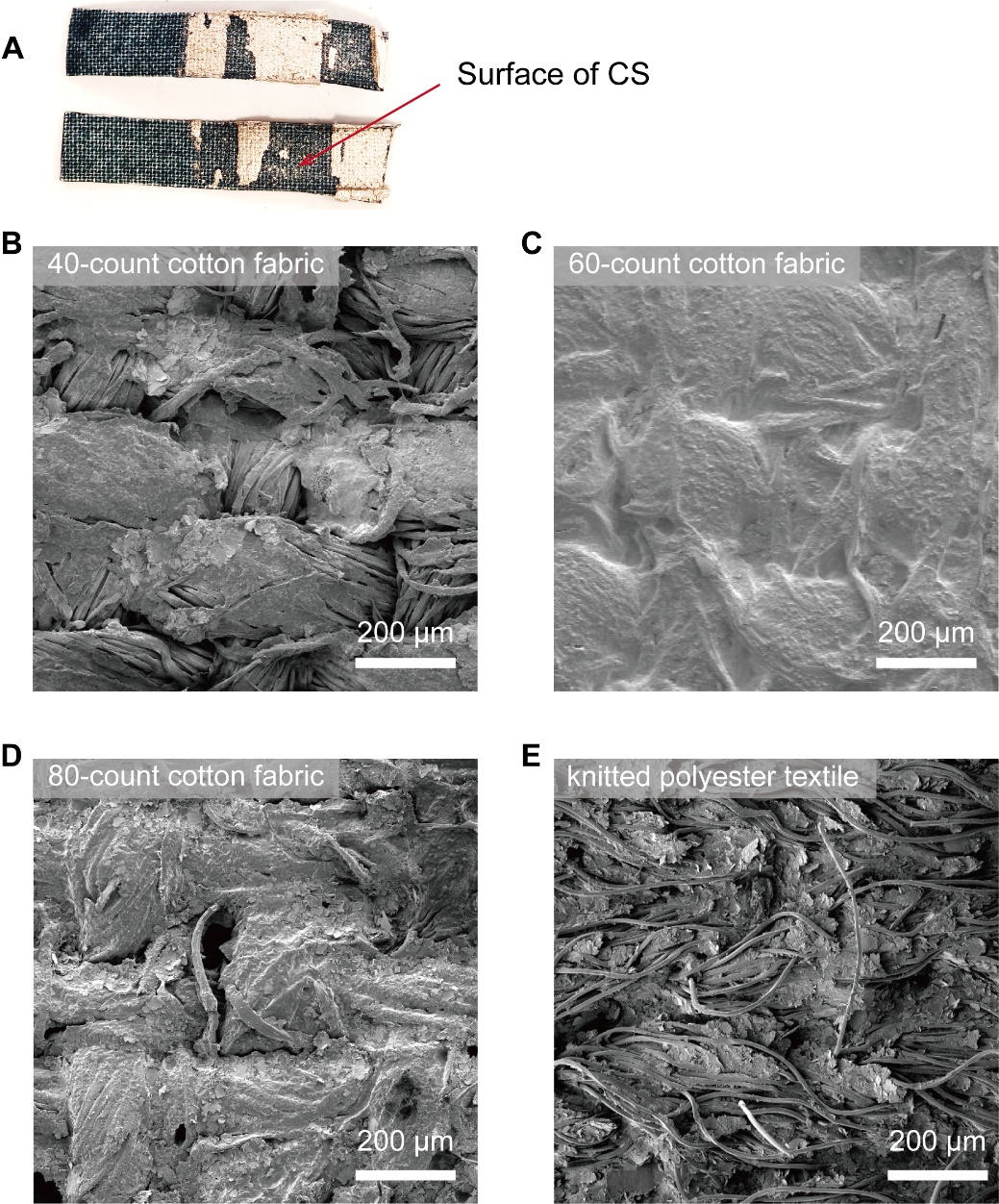


Figure S19. SEM images of the peeled surface of the CS. A) Illustration of the peeled CS surface.(B–D) SEM images of (B) 40-count, (C) 60-count, and (D) 80-count cotton fabrics reveal a similar morphology, showing that the SP filled the voids within the yarns. In the case of the knitted polyester textile-based CS, the SP was also found filled inside the fibers (E).


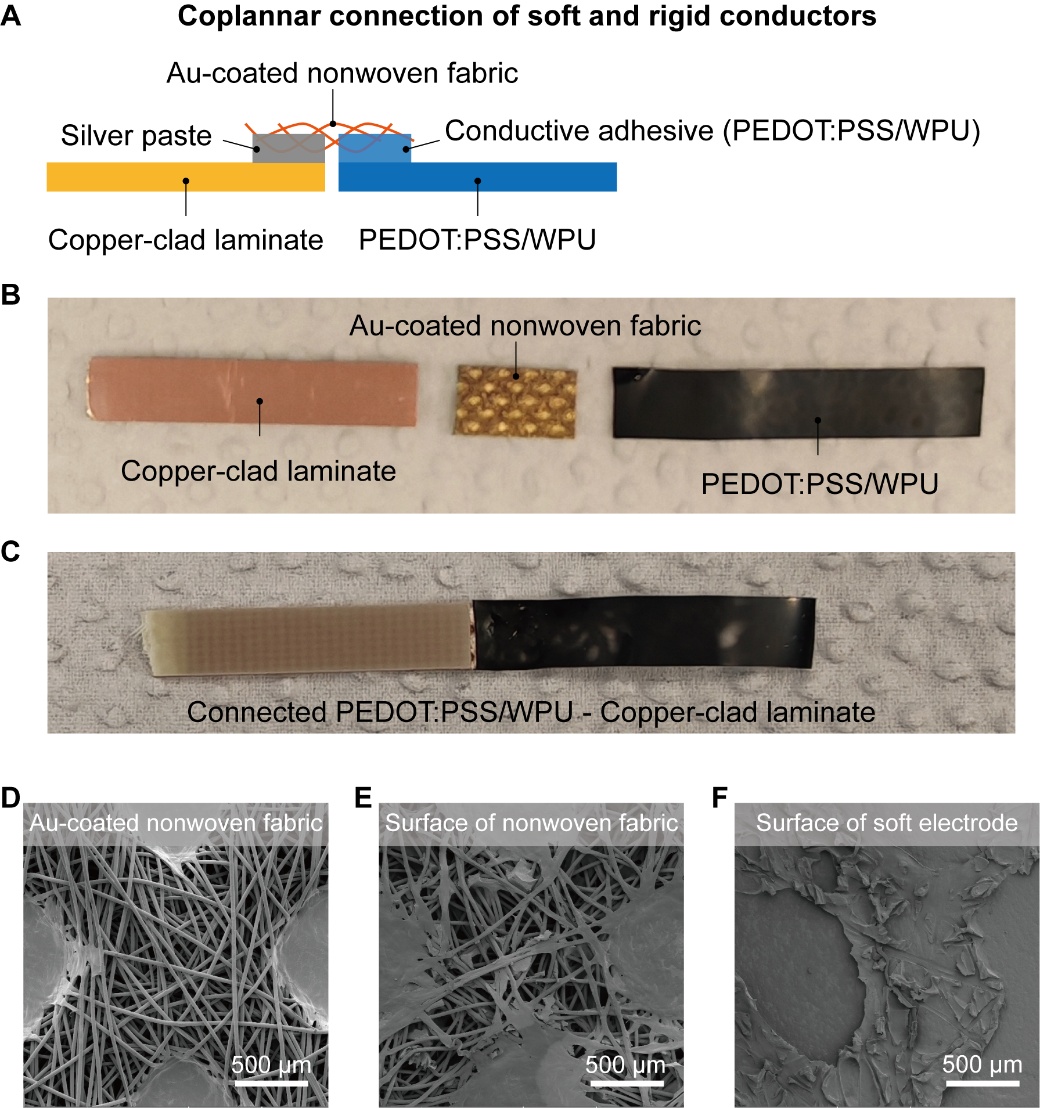


Figure S20. Soft-rigid connection in a coplanar configuration. A) Schematic illustration of connecting the PEDOT:PSS/WPU soft electrode to the copper-clad laminate using a gold-coated, nonwoven fabric-based CS. B) Photographs of the individual components: the copper-clad laminate, the gold-coated nonwoven-based CS, and the PEDOT:PSS/WPU electrode. C) Photograph of the assembled coplanar connection between the PEDOT:PSS/WPU electrode and the copper-clad laminate. D) SEM image of the nonwoven-based CS, E-F) SEM images of (E) the peeled surface of the CS, and (F) the peeled surface of the soft electrode, adhered via the PEDOT:PSS/WPU precursor. The surface of the SP interface is not shown because the nonwoven fabric underwent bulk failure when SP was used as the adhesive.


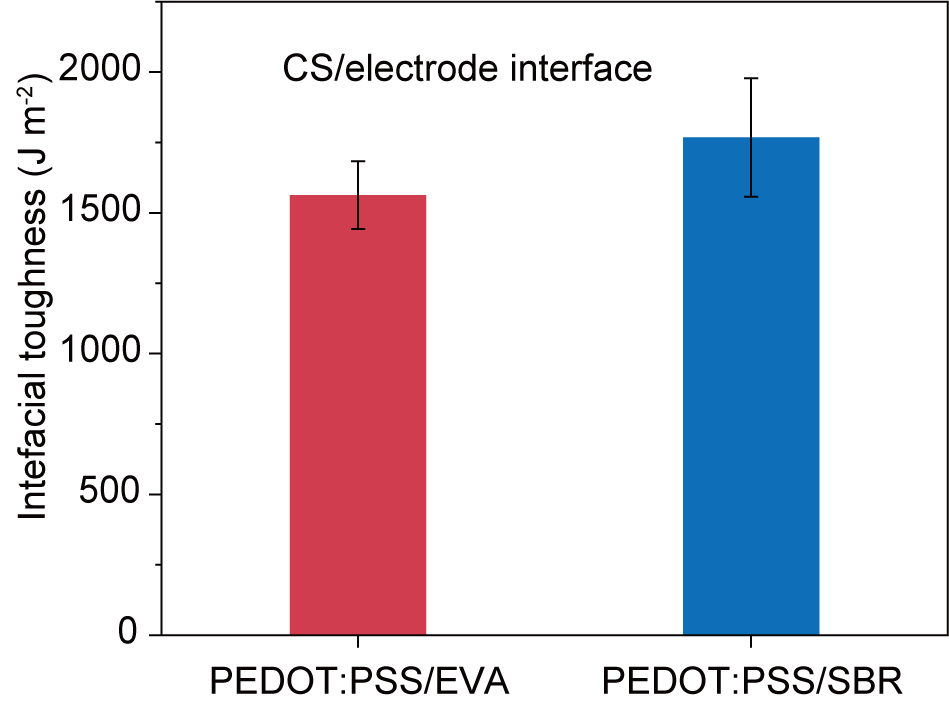


Figure S21. Interfacial toughness of CS on PEDOT:PSS/EVA and PEDOT:PSS/SBR electrodes. The fabrication of these conductive elastomers is similar to that of PEDOT:PSS/WPU, with WPU replaced by EVA and SBR. The weight ratio of PEDOT:PSS to EVA and SBR is maintained at 1:9. Data is presented as mean ± standard deviation from 3 independent measurements.


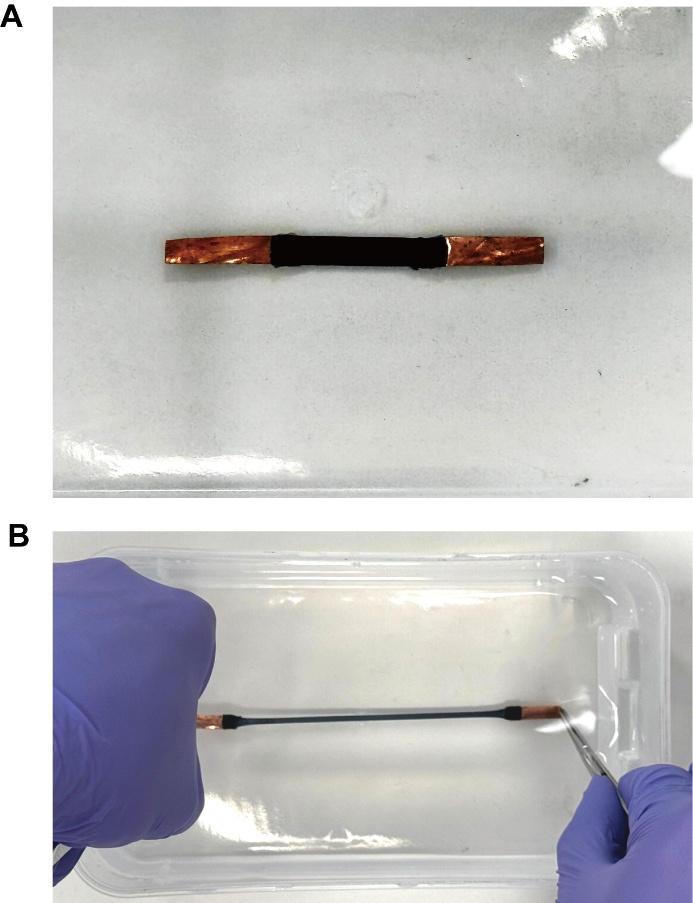


Figure S22. DSMI-connected fully hydrated PEDOT:PSS/WPU-copper can be stretched under water.


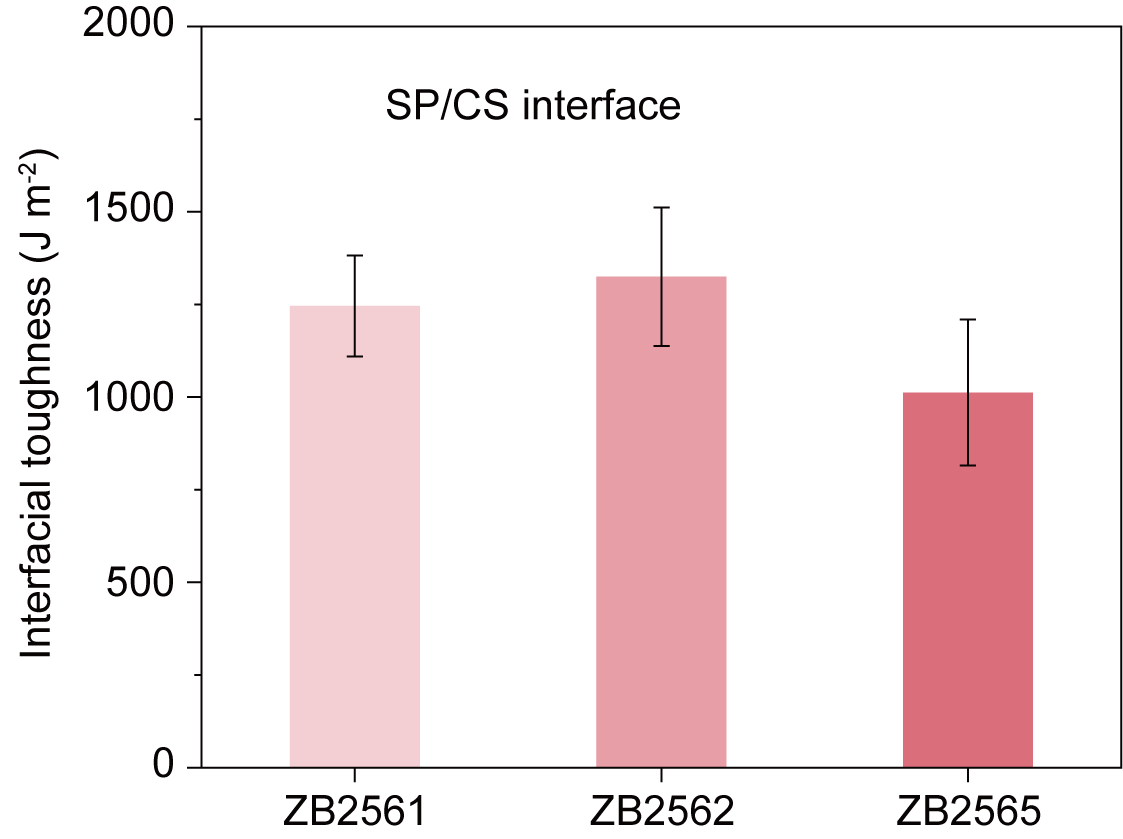


Figure S23. Interfacial toughness of SP on CS. CS (based on 60-count cotton fabric) was first adhered to PEDOT:PSS/WPU before SP application. The SP used contains different resin matrix: ZB2561 (silicon matrix), ZB2562 (two-component epoxy matrix), and ZB2565 (one-component epoxy matrix). Data is presented as mean ± standard deviation from 3 independent measurements.


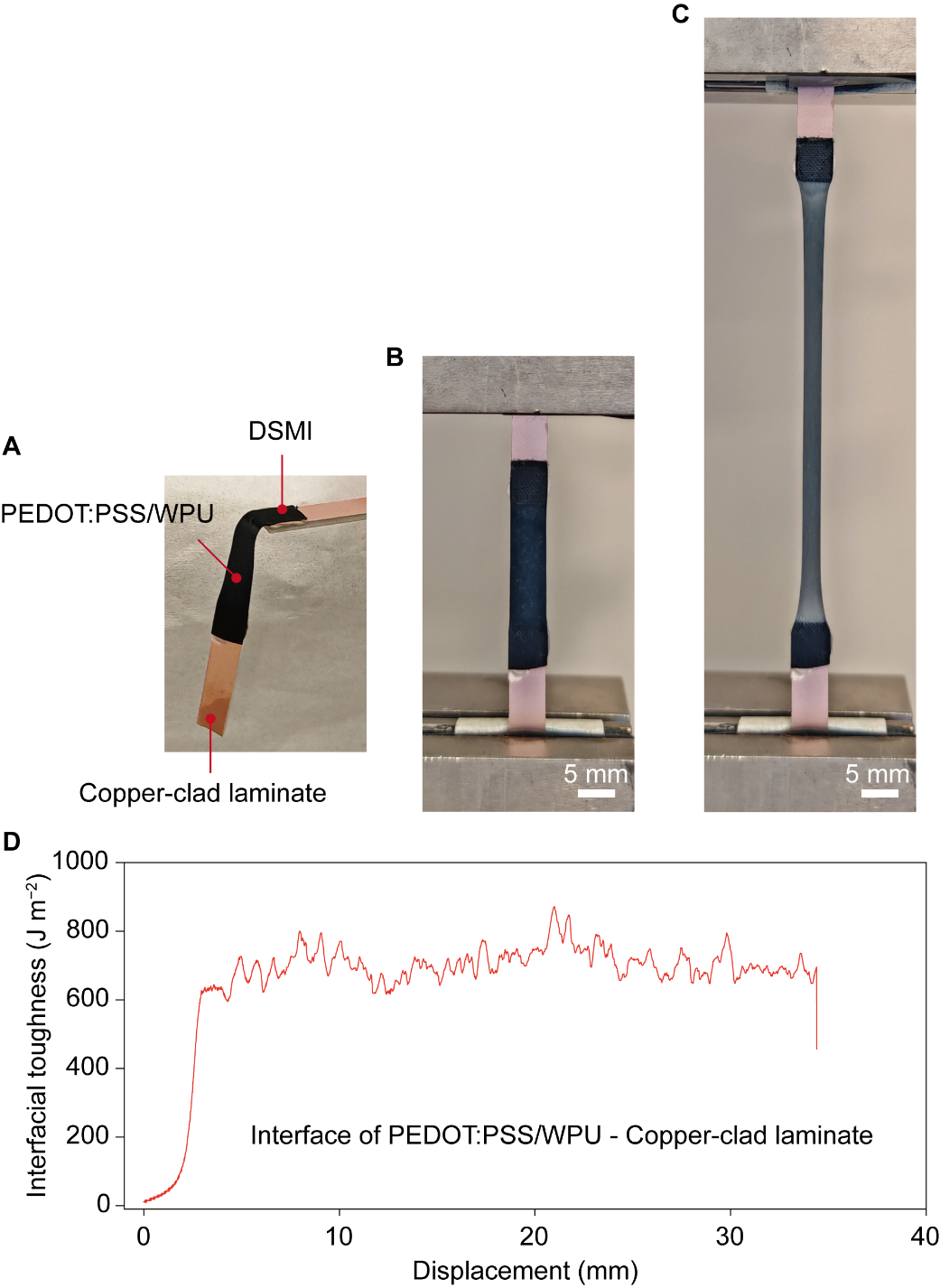


Figure S24. DSMI interface of PEDOT:PSS/WPU with copper-clad laminate. A) Photograph of the sample shows the real rigid nature of copper-clad laminate. B) and C) Relaxing state and Stretching state of the PEDOT:PSS/WPU. D) Interfacial toughness of the DSMI interface.


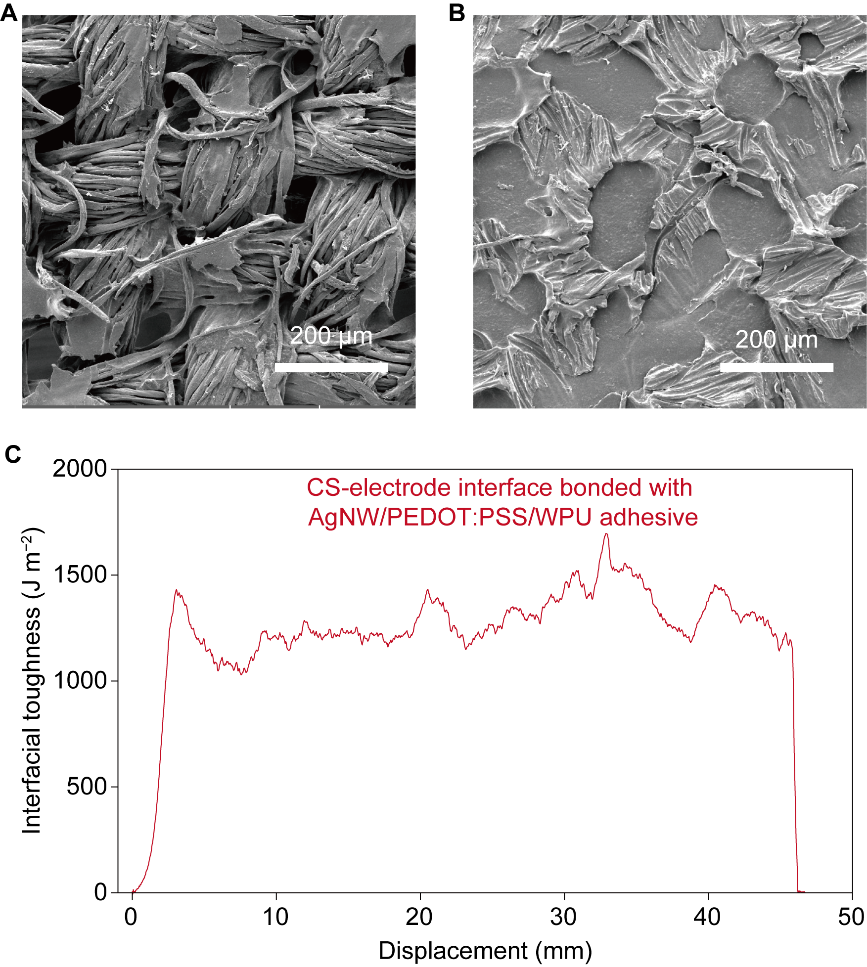


Figure S25. CS-electrode interface with AgNW/PEDOT:PSS/WPU adhesive. A) SEM image of the CS surface peeled from the CS-PEDOT:PSS/WPU interface. (B) SEM image of the PEDOT:PSS/WPU surface peeled from the same interface. The morphologies of the two surfaces match well, indicating cohesive failure of the adhesive. C) Interfacial toughness of the interface. The adhesive formulation consists of 2 g of PEDOT:PSS/WPU precursor mixed with 1 g of AgNW (silver nanowire) aqueous dispersion. The AgNWs have a diameter of 50 nm and a concentration of 10 mg mL^-1^. The electrical conductivity of this AgNW-incorporated adhesive is about 73 S cm^-1^.


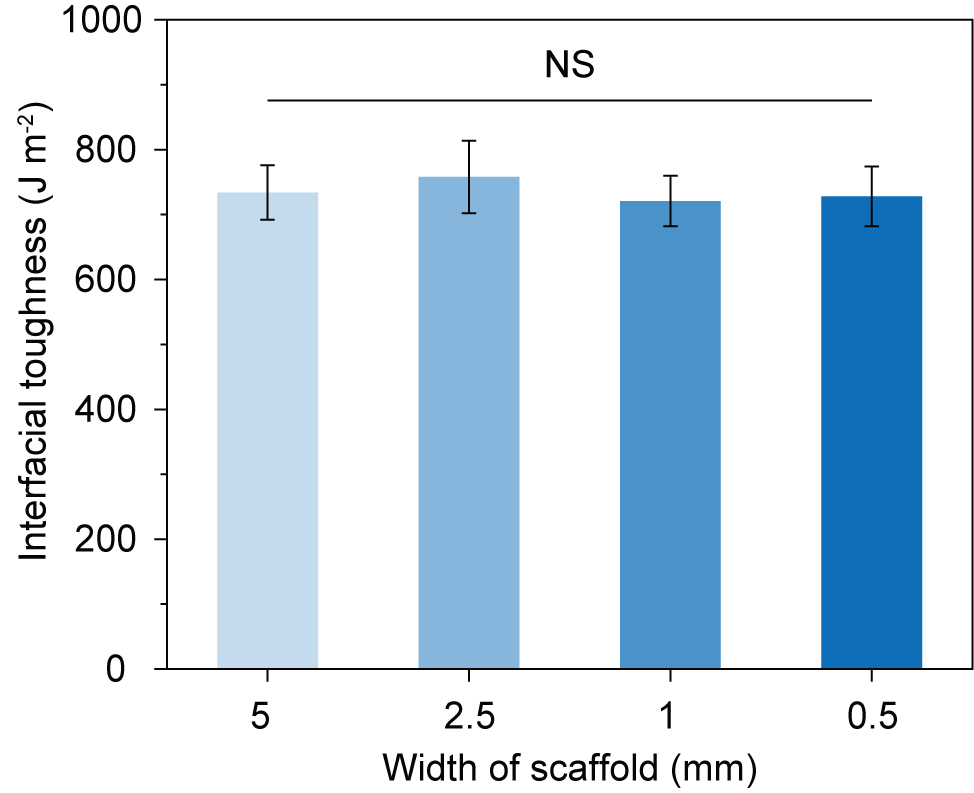


Figure S26. Interfacial toughness of DSMI interfaces with different widths using the 60-count cotton fabric. Data is presented as mean ± standard deviation from 3 independent measurements.


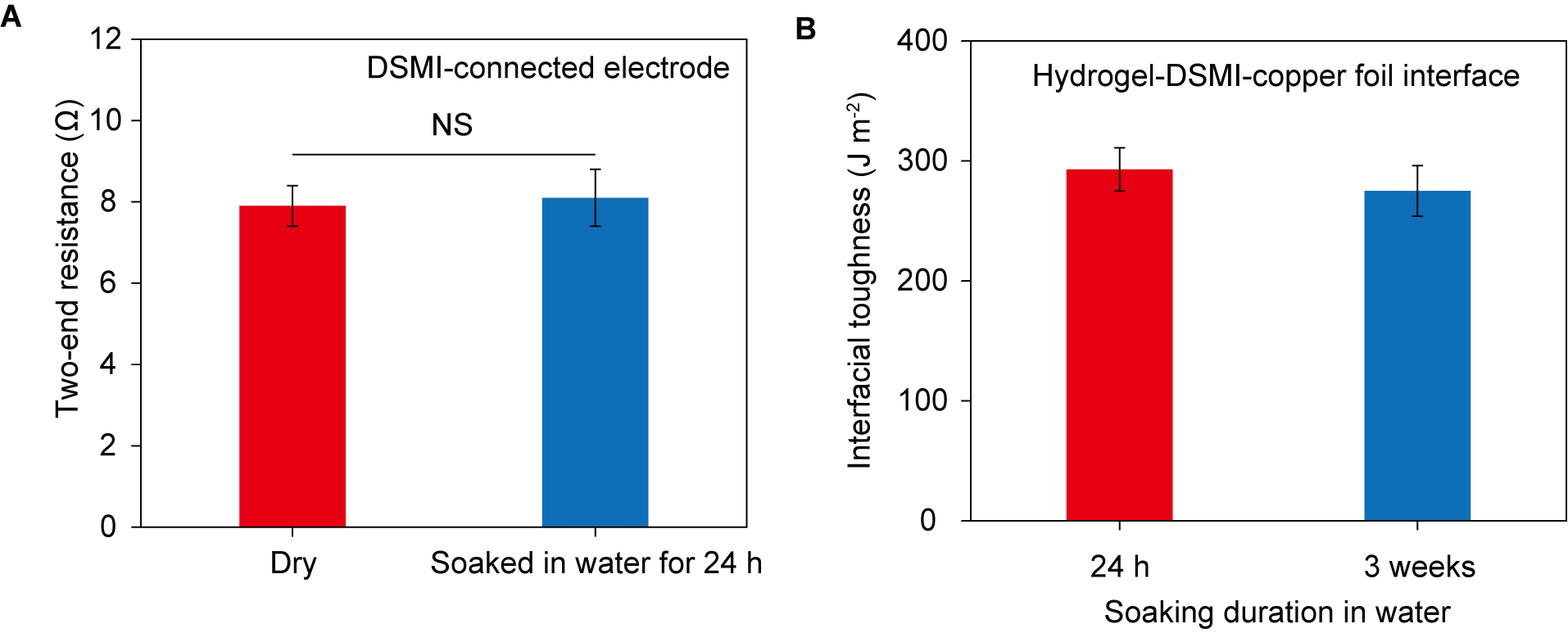


Figure S27. Stability of DSMI-interface in water. A) Water immersion of DMSI-interface does not change the two-end resistance of the electrode. B) Soaking in water for 3 weeks does not comprise the interfacial toughness. Data in panel A and B are presented as mean ± standard deviation from 3 independent measurements.


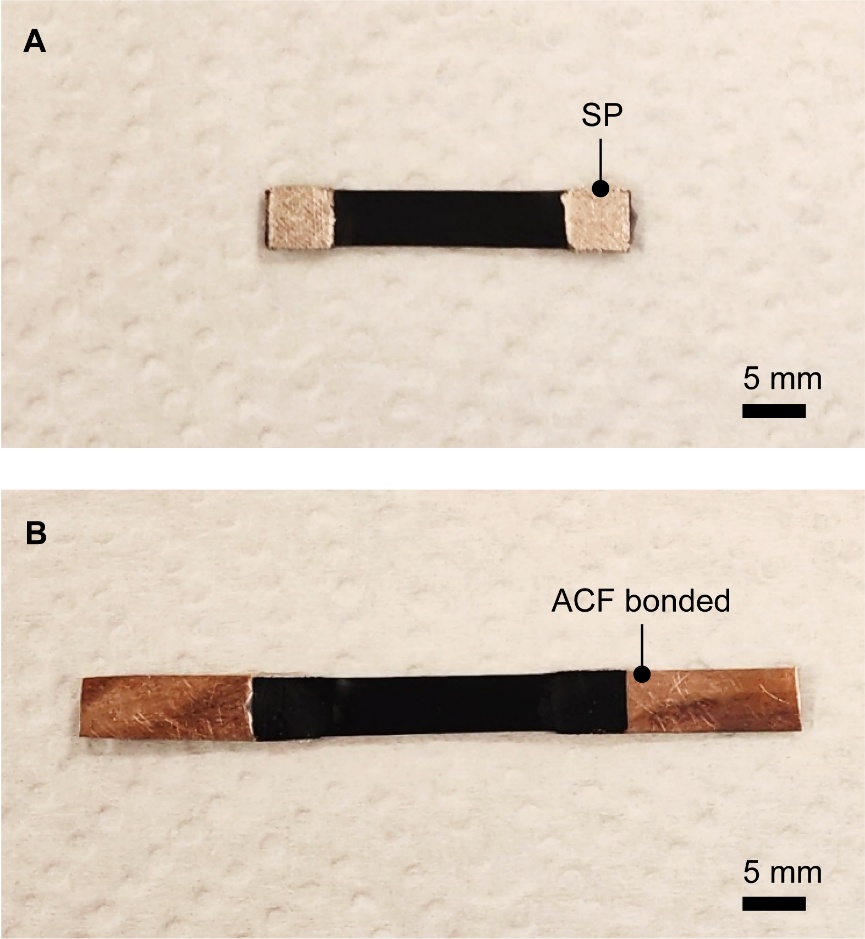


Figure S28. Replaceable electrode preparation. A) SP is applied on the CS and cured. B) ACF is attached to the SP and bonded with copper foil.


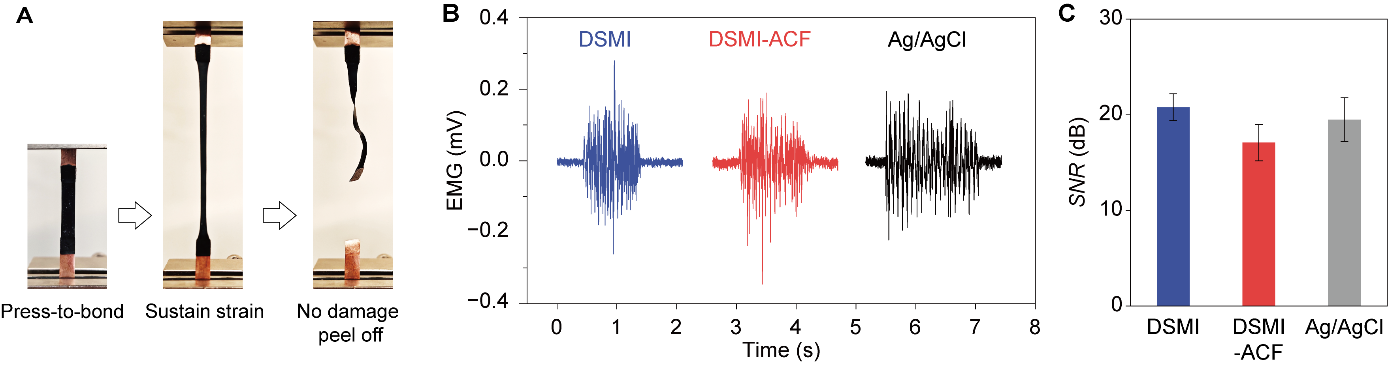


Figure S29. Replaceable electrode based on DSMI-ACF. A) Photographs showing that the DSMI-ACF connection can sustain strain and separate without damage. B) EMG signal recorded by DSMI and DSMI-ACF connected electrode and commercial Ag/AgCl electrode. C) SNR comparison between DSMI and DSMI-ACF connected electrodes and commercial Ag/AgCl electrodes. Data in panel C is presented as mean ± standard deviation from 3 independent measurements.

References:

1. Jiang, Y., et al., *Topological supramolecular network enabled high-conductivity, stretchable organic bioelectronics.* Science, 2022. 375(6587): p. 1411-1417.

2. Zhou, T., et al., *3D printable high-performance conducting polymer hydrogel for all-hydrogel bioelectronic interfaces.* Nature Materials, 2023. 22(7): p. 895-902.

3. Chong, J., et al., *Highly conductive tissue-like hydrogel interface through template-directed assembly.* Nature Communications, 2023. 14(1): p. 2206.

4. Li, T., et al., *Highly robust conductive organo‐hydrogels with powerful sensing capabilities under large mechanical stress.* Advanced Materials, 2024. 36(5): p. 2304145.

5. Tan, P., et al., *Solution-processable, soft, self-adhesive, and conductive polymer composites for soft electronics.* Nature Communications, 2022. 13(1): p. 358.

6. Haque, A.T., et al., *Electrically conductive liquid metal composite adhesives for reversible bonding of soft electronics.* Advanced Functional Materials, 2024. 34(31): p. 2304101.

7. Pozarycki, T.A., et al., *A flexible and electrically conductive liquid metal adhesive for hybrid electronic integration.* Advanced Functional Materials, 2024. 34(31): p. 2313567.

8. Song, Y., et al., *Stretchable and adhesive bilayers for electrical interfacing.* Materials Horizons, 2025.

9. Ai, L., et al., *Tough soldering for stretchable electronics by small-molecule modulated interfacial assemblies.* Nature Communications, 2023. 14(1): p. 7723.

10. Jiang, Y., et al., *A universal interface for plug-and-play assembly of stretchable devices.* Nature, 2023. 614(7948): p. 456-462.

11. Zhao, Y., et al., A universal method for constructing stretchable and conductive connections in flexible electronics. npj Flexible Electronics, 2025. 9(1): p. 63.

12. Zhu, M., et al., *A mechanically interlocking strategy based on conductive microbridges for stretchable electronics.* Advanced Materials, 2022. 34(7): p. 2101339.

13. From 3M Tech Data Sheet. https://www.3m.com/3M/en_US/p/d/b10167835/

14. Yang, Y., et al., *Stretchable and healable conductive elastomer based on PEDOT:PSS/natural rubber for self-powered temperature and strain sensing.* ACS Applied Materials & Interfaces,  2021, 13(12): p. 14599–14611

15. Li, P., et al., *Stretchable and conductive polymer films prepared by solution blending*. ACS Applied Materials & Interfaces, 2015, 7(33):p. 18415–18423

16. Taroni. P., et al., *Toward stretchable self-powered sensors based on the thermoelectric response of PEDOT:PSS/polyurethane blends*. Advanced Functional Materials, 2018, 28(15):p. 1704285

17. Kim. J., et al., *Self-healing, stretchable and recyclable polyurethane-PEDOT:PSS conductive blends*. Materials Horizons, 2024,11:p. 3548-3560
